# Genomic and metabolic characterization of *Trueperella pyogenes* isolated from domestic and wild animals

**DOI:** 10.1128/aem.01725-24

**Published:** 2024-12-31

**Authors:** Gabriela Magossi, Katherine E. Gzyl, Devin B. Holman, T. G. Nagaraja, Raghavendra Amachawadi, Samat Amat

**Affiliations:** 1 Microbiological Sciences Department, North Dakota State University 3323 , Fargo, North Dakota, USA; 2 Lacombe Research and Development Centre, Agriculture and Agri-Food Canada 98668 , Lacombe, Alberta, Canada; 3 College of Veterinary Medicine, Kansas State University 70725 , Manhattan, Kansas, USA; Centers for Disease Control and Prevention, Atlanta, Georgia, USA

**Keywords:** *Trueperella pyogenes*, comparative genomics, antimicrobial resistance, domestic and wild animals, host-and niche-specificity, metabolic profile

## Abstract

*Trueperella pyogenes* is an important bacterial pathogen implicated in infections such as mastitis, metritis, pneumonia, and liver abscesses in both domestic and wild animals, as well as endocarditis and prosthetic joint infections in humans. Understanding the genomic and metabolic features that enable *T. pyogenes* to colonize different anatomical sites within a host and its inter-kingdom transmission and survival is important for the effective control of this pathogen. We employed whole-genome sequencing, phenotype microarrays, and antimicrobial susceptibility testing to identify genomic, metabolic and phenotypic features, and antimicrobial resistance (AMR) genes in *T. pyogenes* recovered from different livestock, companion, and wildlife animals. For comparative genomic analysis, 83 *T. pyogenes* genomes, including 60 isolated in the current study and 23 publicly available genomes were evaluated. These genomes represented *T. pyogenes* strains originating from 16 different body sites of 11 different animal hosts (e.g., cattle, swine, ovine, deer, bison, horse, chamois, and cat). Additionally, 49 *T. pyogenes* isolates (cattle, sheep, deer, swine, and cats) were evaluated for phenotypic AMR using disk diffusion, and for metabolic profiling using the Biology GENIII MicroPlates. The *T. pyogenes* strains were found not to be host- or body site-specific. The presence of conserved virulence genes (*plo* and *fimA*), as well as genotypic and phenotypic AMR may contribute to the ability of *T. pyogenes* to cause infections in livestock, wildlife, and pets. Most of the tested isolates metabolized diverse carbon sources and chemical compounds, suggesting that this metabolic versatility may enhance the survival, competitiveness, and pathogenic potential of *T. pyogenes*.

**IMPORTANCE:**

*Trueperella pyogenes* is an important animal pathogen with zoonotic potential, posing a significant health concern to both animals and humans due to its ability to cause infections across different animal host species and tissues. Current understanding of this pathogen’s adaptability and survival mechanisms is limited. Here, we evaluated the genomic, virulence, metabolic, and antimicrobial resistance (AMR) characteristics of *T. pyogenes* recovered from 16 different body sites of 11 different animal hosts (livestock, companion, and wild animals). We identified multiple AMR and virulence genes that may enable *T. pyogenes* for sustained infection and transmission. Additionally, *T. pyogenes* strains displayed metabolic versatility which could also contribute to its ability to thrive in diverse environments. Understanding the genomic and metabolic, and AMR characteristics that enable *T. pyogenes* to colonize different anatomical sites within a host and its transmission between different animal species is important for the effective control of this pathogen.

## INTRODUCTION

*Trueperella pyogenes* is a Gram-positive pleomorphic, non-motile, non-spore-forming, and facultative anaerobic rod bacterium, formerly classified as *Arcanobacterium pyogenes* (1). This bacterium is an important pathogen implicated in various infections in domestic and wild animals, including metritis, mastitis, and liver abscess in cattle, as well as pneumonia in swine (2). Among livestock hosts, *T. pyogenes* is frequently isolated from cattle (both beef and dairy) and swine infections (3), but sheep, goats, and horses can also be infected. In wild animals, *T. pyogenes*-associated chronic purulent infections (keratoconjunctivitis, brain, and foot abscesses), pneumonia, abscesses, and necrobacillosis are common in captive and free-ranging white-tailed deer (*Odocoileus virginianus*) (4–6). *T. pyogenes* has also been isolated from the lungs of American bison (*Bison bison*) (7), big horn sheep (*Ovis canadensis*) (8), and camels (9), as well as the vaginal discharge of an okapi, and the kidneys of a royal python (10). *T. pyogenes* is also an opportunistic pathogen for companion animals including cats and dogs (11), with clinical manifestations ranging from cystitis and otitis externa to pneumonia and wound infections (12–14). Recently, *T. pyogenes* has emerged as a zoonotic pathogen, causing clinical signs in humans, including endocarditis, ulcers, and sepsis. People working in close contact with farm animals and/or with underlying conditions are at the highest risk for infection with *T. pyogenes* (15–18). Thus, *T. pyogenes* is an important bacterial pathogen that can affect food-producing animals, companion animals, wildlife, and humans.

The genomic and metabolic characteristics of *T. pyogenes* that enable colonization, spread, and infection of such a broad range of hosts and body sites are under explored. Virulence factors could be one of the primary determinants of *T. pyogenes* multi-host and multi-niche colonization (19). The main virulence factor possessed by this species is the cholesterol-dependent cytolysin exotoxin called pyolysin, which is encoded by the gene *plo* and can damage host cell membranes through pore formation (20). Other virulence factors such as collagen-binding protein (*cbpA*), neuraminidases H and P (*nanH* and *nanP*), and types A, C, E, and G fimbriae, encoded by the *fimA*, *fimC*, *fimE*, and *fimG* genes, can contribute to pathogenesis by enabling the colonization of different mucosal niches (5, 21–23). Niche-specific genetic, metabolic, and phenotypic evolution have been relatively well documented in some bacterial species, particularly *Escherichia coli* (24–27), and *Lactobacillus* spp. (28–30). Thus, it is likely that *T. pyogenes* strains in different mammalian hosts and different body sites within an animal may have evolved both genetically and phenotypically to adapt to these different niches. However, there are limited data available on the genomic (diverse pangenome and intergenotypic variability), phenotypic (metabolic pathways), and ecotypic (host-/niche-specificity) diversity among *T. pyogenes* present in different hosts and body sites.

Resistance to antimicrobials commonly used in animal production systems, such as the tetracyclines (31), trimethoprim-sulfamethoxazole, and macrolides-lincosamides-streptogramin B (MLS_B_) has been reported in *T. pyogenes* isolates (32). However, whether the antimicrobial susceptibility of *T. pyogenes* strains varies depending on the host and niche origin is unknown. Given that *T. pyogenes* has the potential to transfer among domestic (including livestock and pets) and wild animals, and from animals to humans, it is important from a One Health perspective to characterize antimicrobial resistance (AMR) in *T. pyogenes* originating from different hosts. Thus, the objectives of this study were to: (i) isolate *T. pyogenes* from different animal species (domestic and wild) and infection body sites, (ii) perform comparative genomic and metabolic analyses to identify genotypic and phenotypic characteristics associated with host-/niche-specificity, and (iii) characterize and compare genotypic and phenotypic AMR profiles among *T. pyogenes* isolated from different animal hosts and body sites.

## RESULTS

### *T. pyogenes* strains origin

The 60 *T. pyogenes* strains used in the present study were isolated from cattle (*n* = 49), swine (*n* = 3), sheep (*n* = 3), deer (*n* = 3), bison (*n* = 1), and cats (*n* = 1), with strains most frequently originating from ruminal tissues (*n* = 24) and lungs (*n* = 22). One of the three swine-origin isolates was obtained from ATCC (strain ATCC 19411). Additionally, 23 publicly available genomes from cattle (*n* = 9), swine (*n* = 8), water buffalo (*n* = 2), dog (*n* = 1), goat (*n* = 1), chamois (*n* = 1), and horse (*n* = 1) were included (Table 1).

**TABLE 1:** *Trueperella pyogenes* genome assemblies and isolates

Accession number	Isolate ID	Animal host	Anatomical body site	Source	AST	Metabolite
GCF_000599565	MS249	Cattle	Uterus	NCBI	No	No
GCF_000612055	TP6375	Cattle	Uterus	NCBI	No	No
GCF_001281085	2012CQ-ZSH	Goat	Lung	NCBI	No	No
GCF_002071825	UFV1	Cattle	Uterus	NCBI	No	No
GCF_002762575	Bu5	Water buffalo	Wound	NCBI	No	No
GCF_003055835	Arash114	Water buffalo	Uterus	NCBI	No	No
GCF_003076295	TP4479	Swine	N/A	NCBI	No	No
GCF_003076315	TP-2849	Swine	Lung	NCBI	No	No
GCF_003971445	TP1	Cattle	Lung	NCBI	No	No
GCF_003971465	TP2	Cattle	Joint	NCBI	No	No
GCF_003971485	TP3	Swine	Lung	NCBI	No	No
GCF_003971545	TP4	Swine	Lung	NCBI	No	No
GCF_004123715	SH03	Swine	Lung	NCBI	No	No
GCF_004009995.2	SH02	Swine	Lung	NCBI	No	No
GCF_004564055	SH01	Swine	Lung	NCBI	No	No
GCF_012109075	jx18	Swine	Lung	NCBI	No	No
GCF_024741855	22/KM0800	Cattle	Wound	NCBI	No	No
GCF_024803545	EMSSI21	Cattle	Wound	NCBI	No	No
GCF_024803565	EMSSI54	Cattle	Wound	NCBI	No	No
GCF_024803585	EMSSI48	Cattle	Wound	NCBI	No	No
GCF_030078095	09KM1269	Dog	N/A	NCBI	No	No
GCF_030078115	13KM1326	Horse	N/A	NCBI	No	No
GCF_030078145	13OD0707	Chamois	N/A	NCBI	No	No
GCF_040763425.1	ATCC_19411	Swine	N/A	This study	No	Yes
GCF_040763385.1	93CBB	Cattle	Vaginal swab	This study	Yes	Yes
GCF_040763445.1	51CBC	Cattle	Vaginal swab	This study	Yes	Yes
GCF_040763435.1	4618_1	Cattle	Abscess	This study	Yes	Yes
GCF_040763405.1	23_564_0001_02	Cattle	Liver	This study	No	Yes
GCF_040763485.1	23_50711_2	Cattle	Lung	This study	No	Yes
GCF_040763505.1	23_4130	Cattle	Lung	This study	Yes	Yes
GCF_040763555.1	23_30761_2	White-tailed deer	Lung	This study	Yes	Yes
GCF_040763575.1	22_7871	Sheep	Mammary	This study	Yes	Yes
GCF_040763585.1	22_5896	Cattle	Lung	This study	Yes	Yes
GCF_040763625.1	22_5293	Cattle	Foot	This study	Yes	Yes
GCF_040763645.1	22_5283	Cattle	Lung	This study	Yes	Yes
GCF_040763655.1	22_4838	Cattle	Lung	This study	Yes	Yes
GCF_040763665.1	22_4813	Cattle	Lung	This study	No	Yes
GCF_040763715.1	22_2570	Cattle	Milk	This study	No	Yes
GCF_040763675.1	22_2565	Bison	Lung	This study	No	No
GCF_040763755.1	22_2564	Cattle	Peri fluid	This study	No	Yes
GCF_040763745.1	22_11656	Cattle	Lung	This study	Yes	Yes
GCF_040763785.1	22_11177	Cattle	Lung	This study	Yes	Yes
GCF_040763805.1	22_11067	Sheep	Umbilicus swab	This study	Yes	Yes
GCF_040763795.1	22_10600	Cat	Nasal swab	This study	Yes	Yes
GCF_040763835.1	21_2041	White-tailed deer	Lung	This study	Yes	Yes
GCF_040763855.1	2023_4_80	Cattle	Rumen tissue	This study	Yes	Yes
GCF_040763885.1	2023_3_72	Cattle	Rumen tissue	This study	Yes	Yes
GCF_040763905.1	2023_3_71	Cattle	Rumen tissue	This study	No	No
GCF_040763955.1	2023_3_67	Cattle	Rumen tissue	This study	No	Yes
GCF_040763945.1	2023_3_60	Cattle	Rumen tissue	This study	No	No
GCF_040763915.1	2023_3_4	Cattle	Rumen tissue	This study	Yes	Yes
GCF_040763965.1	2023_3_235	Cattle	Rumen tissue	This study	Yes	Yes
GCF_040764015.1	2023_3_175	Cattle	Rumen tissue	This study	No	Yes
GCF_040764025.1	2023_3_174	Cattle	Rumen tissue	This study	No	No
GCF_040764065.1	2023_3_167	Cattle	Rumen tissue	This study	Yes	No
GCF_040764005.1	2023_3_164	Cattle	Rumen tissue	This study	No	Yes
GCF_040764085.1	2023_3_163	Cattle	Rumen tissue	This study	No	Yes
GCF_040764095.1	2023_3_119	Cattle	Rumen tissue	This study	No	No
GCF_040764145.1	2023_3_111	Cattle	Rumen tissue	This study	No	No
GCF_040764165.1	2022_1_96	Cattle	Rumen tissue	This study	No	No
GCF_040764215.1	2022_1_94	Cattle	Rumen tissue	This study	No	Yes
GCF_040764205.1	2022_1_79	Cattle	Rumen tissue	This study	No	Yes
GCF_040764185.1	2022_1_77	Cattle	Rumen tissue	This study	Yes	No
GCF_040764245.1	2022_1_75	Cattle	Rumen tissue	This study	Yes	No
GCF_040764265.1	2022_1_30	Cattle	Rumen tissue	This study	Yes	Yes
GCF_040764285.1	2022_1_29	Cattle	Rumen tissue	This study	Yes	Yes
GCF_040764325.1	2022_1_27	Cattle	Rumen tissue	This study	No	Yes
GCF_040764305.1	2022_1_14	Cattle	Rumen tissue	This study	No	Yes
GCF_040764335.1	8896	Swine	Lung	This study	Yes	No
GCF_040764345.1	7762	Cattle	Lung	This study	Yes	No
GCF_040764415.1	1510	Cattle	Lung	This study	Yes	No
GCF_040764445.1	1494	White-tailed deer	Lung	This study	Yes	No
GCF_040764425.1	1446	Cattle	Lung	This study	Yes	No
GCF_040764405.1	1409	Cattle	Lung	This study	Yes	No
GCF_040764435.1	1361	Cattle	Lung	This study	Yes	Yes
GCF_040764535.1	1355	Ovine	Lung	This study	Yes	No
GCF_040764515.1	1259	Cattle	Lung	This study	Yes	No
GCF_040764585.1	315	Cattle	Liver abscess	This study	Yes	No
GCF_040764525.1	311	Cattle	Liver abscess	This study	Yes	No
GCF_040764505.1	306	Cattle	Liver abscess	This study	Yes	No
GCF_040763545.1	23_2752	Swine	Lung	This study	Yes	Yes
GCF_040764105.1	2023_3_104	Cattle	Rumen tissue	This study	No	Yes
GCF_040764385.1	5861	Cattle	Lung	This study	Yes	No

### Genome assembly and annotation

Of the 60 *T. pyogenes* genomes that we sequenced (59 newly isolated strains and 1 from ATCC), the average genome size was 2,278,603 ± 6,648 (SE) bp, G + C content was 59.6 ± 0.01%, and the number of coding sequences was 2,026 ± 6.51 (Table S1). These genomes contained 46 ± 0.05 tRNA genes, 3 rRNA genes, and 814 ± 8.6 unclassified hypothetical protein coding regions on average. A total of 54 strains (90.0%) had the CRISPR-associated nuclease/helicase gene *cas3*, and 56 strains (93.3%) displayed at least one repeat region in their genomes (Table S1).

### Comparative genome analysis

**Fig 1:**
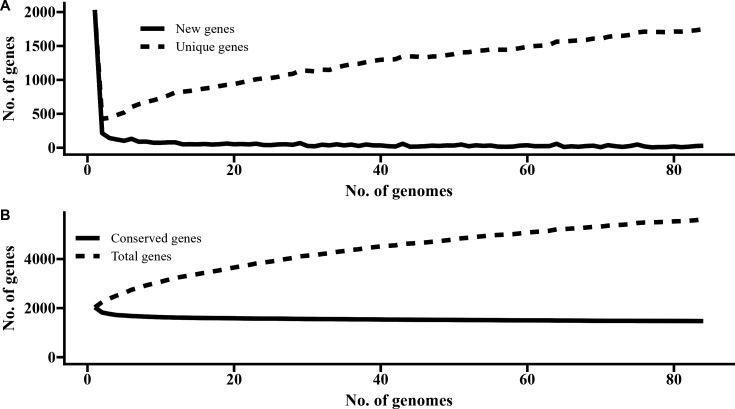
Distribution of (**A**) unique and (**B**) conserved genes in the pangenome of *Trueperella pyogenes* (*n* = 83).

**Fig 2:**
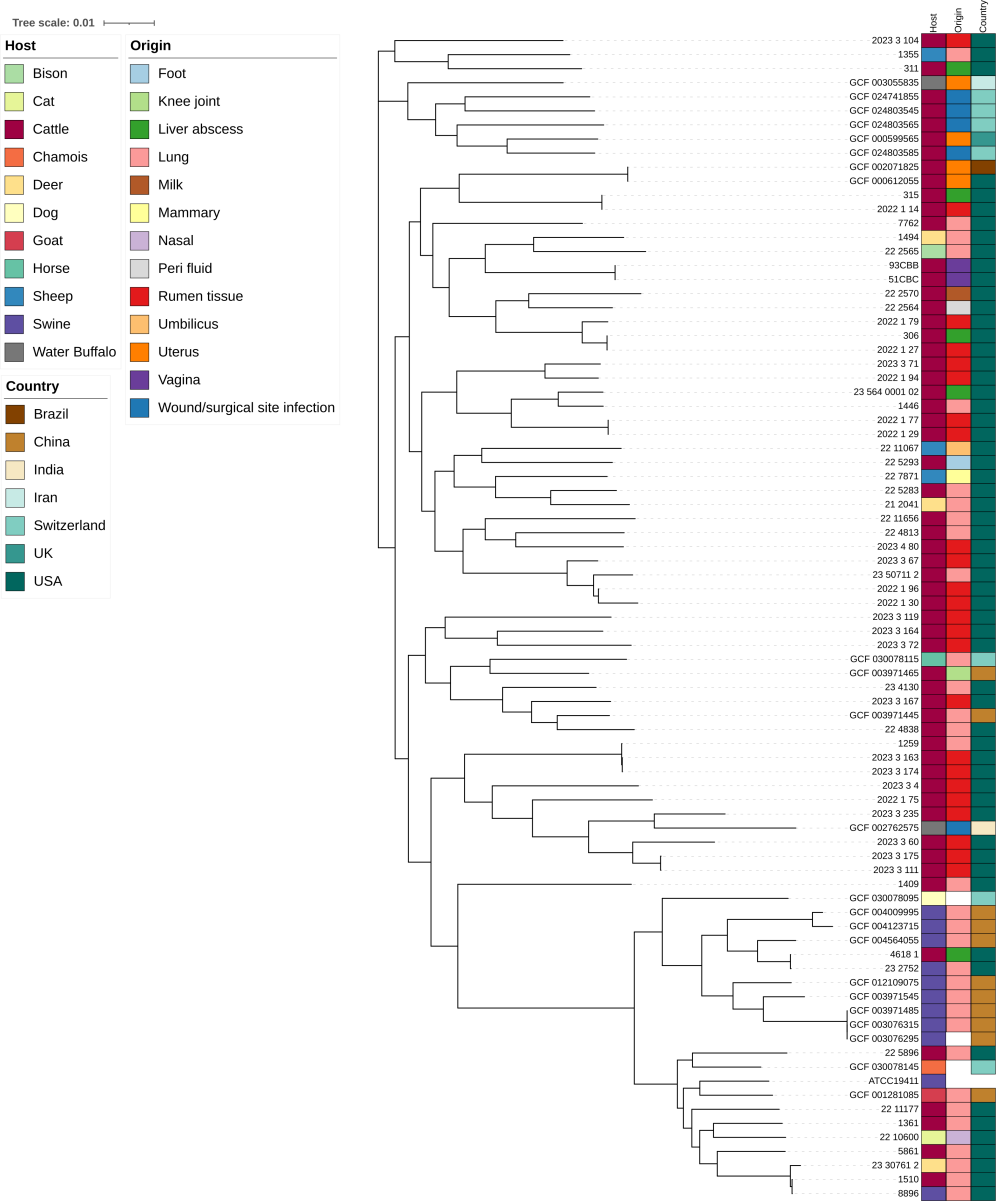
Maximum likelihood phylogenetic tree of *Trueperella pyogenes* isolates obtained from this study (*n* = 60) and publicly available genomes (*n* = 23). Phylogeny was inferred from the alignment of 1,470 core genes. The horizontal scale bar represents substitutions per nucleotide.

In addition to the 60 strains that we sequenced, 23 publicly available genome sequences were included in our comparative genomic analysis to gain more comprehensive insight into *T. pyogenes*. The pangenome of 83 *T. pyogenes* genomes contained 5,612 genes, and of these, 1,470 were core genes that were shared among all genomes. The remaining 4,142 genes were accessory genes that can be divided into softcore (95–99% of genomes; *n* = 155), shell (15–95% of genomes; *n* = 609), and cloud (0–15% of genomes; *n* = 3,378) genes. *T. pyogenes* appears to have an open pangenome as there was no plateau in the number of new genes added to the pangenome as more genomes were included (Fig. 1). Phylogenetic analysis showed that there were several clades with cattle and swine isolates largely clustering separately from each other (Fig. 2). Moreover, there was no distinct clustering of genomes based on the anatomical body site or country of origin. There were 20 genomes with an average nucleotide identity (ANI) of >99.99% with at least one other genome, and 22 with an ANI between 99.5% and 99.99% (Table S2). Based on the phylogenetic tree and their ANI values, there were certain *T. pyogenes* isolates recovered from different host species that appeared to be nearly identical (ANI > 99.99%). This included the isolates 8896 and 1510, which were recovered from the lungs of a pig and cattle in 2016 and 2023, respectively, at the NDSU-VDL. *T. pyogenes* 4618_1 (cattle abscess) and 23_2752 (swine lung) were also isolated in 2016 and 2023, respectively, at the NDSU-VDL, suggesting possible inter-species transfer (Table S2). Consequently, it appears that these strains can colonize and cause disease in both host species, in addition to persisting in the animal population/environment for long periods of time. Additionally, some isolates could potentially be more pathogenic or virulent, such as isolates 2022_1_29 and 2022_1_77 from cattle ruminal tissue. These isolates share eight antimicrobial resistance genes (ARGs), resulting in genotypical AMR to six antimicrobial classes (i.e., aminoglycosides, biocides, glycopeptides, MLS_B_, sulfonamides, and tetracyclines) and phenotypical resistance to clindamycin (lincosamide), gentamicin (aminoglycoside), and tetracycline (Table S4). They are also genetically similar, with ANI > 99.99% (Table S2), suggesting that they are the same strain of *T. pyogenes*.

### Virulence genes and genotypes

**Fig 3:**
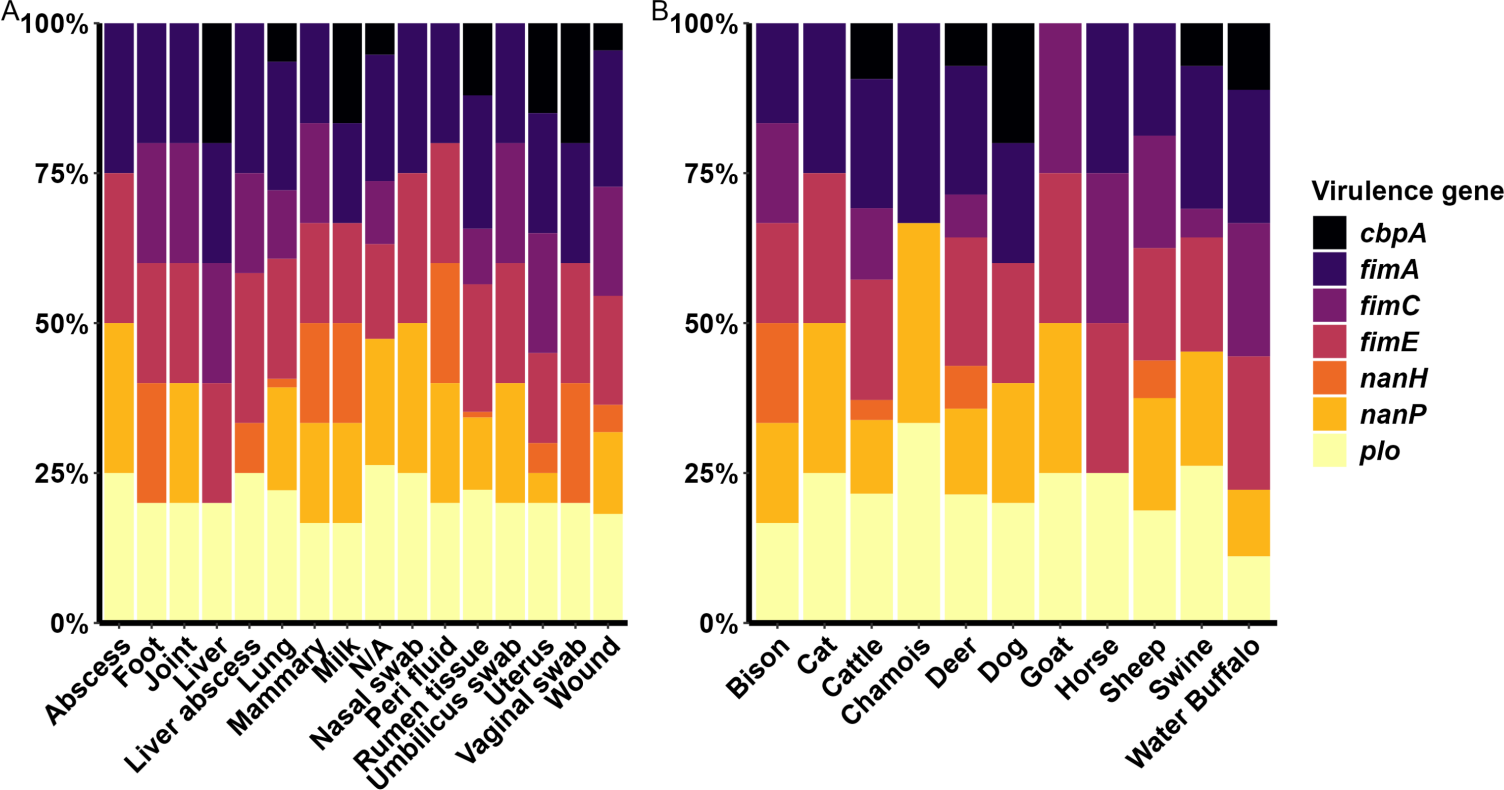
Distribution and proportion of virulence genes in the genomes of *Trueprella pyogenes* (*n* = 83) isolates by (**A**) infection site and (**B**) host species.

The 83 *T. pyogenes* genomes investigated in this study were screened for the presence of seven known virulence genes. The most prevalent virulence genes were *plo* (98.8%), *fimA* (97.6%), *fimE* (90.4%), and *nanP* (63.9%), followed by *fimC* (51.8%), *cbpA* (41%), and *nanH* (14.5%) (Fig. 3). Based on the combination of virulence genes, the genomes were categorized into 20 genotypes. The predominant genotypes were VIII with the *plo*, *fimA*, *fimC*, *fimE*, and *nanP*, genes (14.5%), XVI with the *plo*, *fimA*, *fimE*, *nanP* genes (14.5%), XI with the *plo*, *fimA*, *fimE*, *nanP*, and *cbpA* genes (9.6%), VI with the *plo*, *fimA*, *fimC, fimE*, and *cbpA* genes (7.2%), and XIII with *plo*, *fimA*, *fimC*, and *fimE* (7.2%) (Table S3). The proportion of virulence genes across different anatomical body sites or among animal host species did not follow any detectable patterns (Fig. 3).

### ARG profiles

**Fig 4:**
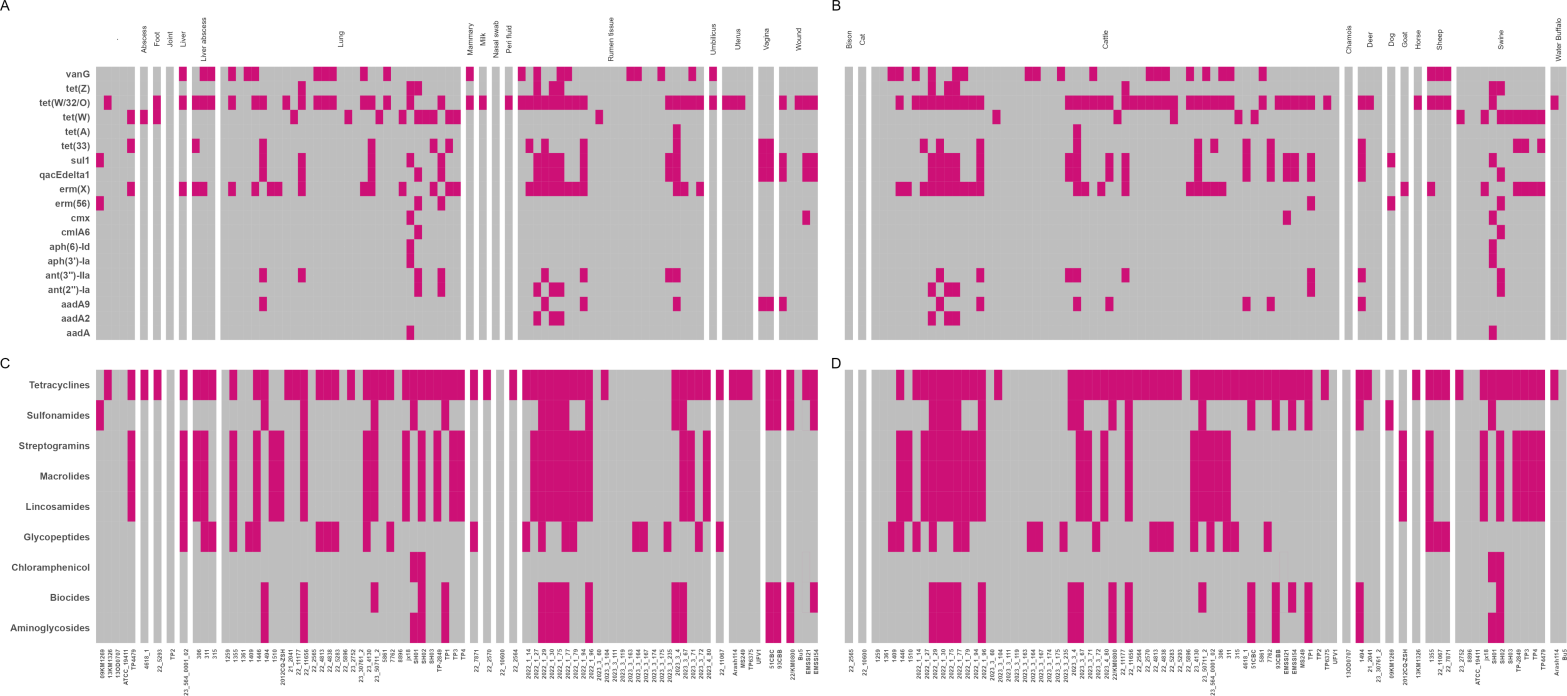
Antimicrobial resistance genes (ARGs) in *Trueperella pyogenes* isolates (*n* = 83) by (**A**) body site, and (**B**) host species, and antimicrobial class for which the ARGs confer resistance to (**C**) anatomical body site, and (**D**) host species.

There were 19 different ARGs identified among the 83 *T. pyogenes* genomes (Table 2). The ribosomal protection protein gene *tet*(W/32/O) conferring resistance to the tetracyclines was the most prevalent ARG (53% of genomes), followed by *erm*(X) (31.3%), *vanG* (25.3%), *sul1* (21.7%), and *qacEdelta1* (20.5%) (Table 2). Moreover, 19 *T. pyogenes* genomes (22.9%) did not carry any ARGs, including the ATCC 19411 reference strain. The ARGs identified confer resistance to many different antimicrobial classes, namely tetracyclines (69.9%), MLS_B_ (31.3%), glycopeptides (25.3%), sulfonamides (21.7%), biocides (20.5%), aminoglycosides (18.1%), and phenicols (3.6%) (Table 3). When the *T. pyogenes* isolates were grouped based on body site origin, the liver and liver abscess isolates had a similar ARG profile with genes encoding resistance to tetracyclines, aminoglycosides, and MLS_B_. This was similar to the ARG profiles of multiple ruminal tissue-associated isolates (Fig. 4). Several isolates encoded multiple ARGs that appeared to be on the same contig together with certain mobile genetic element genes (Fig. S1). *T. pyogenes* 2023_3_67, isolated from cattle ruminal tissue, carried eight ARGs, including *erm*(X) and *tet*(33) which were co-located on an 8,048 bp contig with a *repA* gene (replication initiation) and *ant(3″)-IIa*, *qacEdelta1*, and *sul1* which were linked with an integrase (*int*) gene on a 4,214 bp contig. In two other cattle ruminal tissue isolates (2022_1_29 and 2022_1_75), *sul1*, *qacEdelta1*, *aadA2*, and *ant(2'')-Ia* were found together on a 5,857 bp contig also containing the plasmid-associated genes *parA* (partition) and *int*. The *sul1* and *qacEdelta1* genes were linked together in all isolates carrying both ARGs. Additionally, isolates 2022_1_29 and 2022_1_77, also recovered from cattle ruminal tissue, shared eight ARGs conferring resistance to six antimicrobial classes (i.e., aminoglycosides, biocides, glycopeptides, MLS_B_, sulfonamides, and tetracyclines). They were also genetically very similar (ANI > 99.99%; Table S2), suggesting that they are the same strain.

**TABLE 2:** Distribution (%) of antimicrobial resistance genes in the genomes of *Trueperella pyogenes* from different host species and anatomical body sites (*n* = 83)

		aadA	aadA2	aadA9	ant(2″)-Ia	ant(3″)-IIa	aph(3′)-Ia	aph(6)-Id	cmlA6	cmx	erm(X)	msrA	qacEdelta1	sul1	tet(33)	tet(A)	tet(W)	tet(W/32/O)	tet(Z)	vanG
Host species	Bison	0	0	0	0	0	0	0	0	0	0	0	0	0	0	0	0	0	0	0
	Cattle	0	3 (5.2)	6 (10.3)	4 (6.9)	6 (10.3)	0	0	0	1 (1.7)	19 (32.8)	1 (1.7)	15 (25.9)	15 (25.9)	8 (13.8)	1 (1.7)	5 (8.7)	36 (62.1)	4 (6.9)	18 (31)
	Water buffalo	0	0	0	0	0	0	0	0	0	0	0	0	0	0	0	0	1 (50)	0	0
	Dog	0	0	0	0	0	0	0	0	0	0	0	0	1 (100)	0	0	0	0	0	0
	Goat	0	0	0	0	0	0	0	0	0	1 (100)	0	0	0	0	0	0	0	0	0
	White-tailed deer	0	0	1 (33.3)	0	1 (33.3)	0	0	0	0	0	0	1 (33.3)	1 (33.3)	1 (33.3)	0	0	2 (66.7)	0	0
	Chamois	0	0	0	0	0	0	0	0	0	0	0	0	0	0	0	0	0	0	0
	Horse	0	0	0	0	0	0	0	0	0	0	0	0	0	0	0	0	1 (100)	0	0
	Cat	0	0	0	0	0	0	0	0	0	0	0	0	0	0	0	0	0	0	0
	Sheep	0	0	0	0	0	0	0	0	0	1 (100)	0	0	0	0	0	0	3 (100)	0	3 (100)
	Swine	1 (9.1)	0	0	1 (9.1)	1 (9.1)	1 (9.1)	1 (9.1)	1 (9.1)	1 (9.1)	5 (45.5)	0	1 (9.1)	1 (9.1)	3 (27.3)	0	8 (72.7)	1 (9.1)	2 (18.2)	0
Anatomical body site	Abscess	0	0	0	0	0	0	0	0	0	0	0	0	0	0	0	1 (100)	0	0	0
	Foot	0	0	0	0	0	0	0	0	0	0	0	0	0	0	0	1 (100)	1 (100)	0	0
	Joint	0	0	0	0	0	0	0	0	0	0	0	0	0	0	0	0	0	0	0
	Liver	0	0	0	0	0	0	0	0	0	1 (100)	0	0	0	0	0	0	1 (100)	0	1 (100)
	Liver abscess	0	0	0	0	0	0	0	0	0	2 (66.7)	0	0	0	1 (33.3)	0	0	3 (100)	0	2 (66.7)
	Lung	1 (3.2)	0	1 (3.2)	1 (3.2)	3 (9.7)	1 (3.2)	1 (3.2)	1 (3.2)	1 (3.2)	11 (35.5)	0	5 (16.1)	5 (16.1)	4 (12.9)	0	9 (29)	13 (41.9)	3 (9.7)	8 (25.8)
	Mammary	0	0	0	0	0	0	0	0	0	0	0	0	0	0	0	0	1 (100)	0	1 (100)
	Milk	0	0	0	0	0	0	0	0	0	0	0	0	0	0	0	0	1 (100)	0	0
	N/A	0	0	0	0	0	0	0	0	0	1 (20)	0	0	1 (20)	1 (20)	0	1 (20)	1 (20)	0	0
	Nasal swab	0	0	0	0	0	0	0	0	0	0	0	0	0	0	0	0	0	0	0
	Peri fluid	0	0	0	0	0	0	0	0	0	0	0	0	0	0	0	0	1 (100)	0	0
	Rumen tissue	0	3 (12.5)	3 (12.5)	3 (12.5)	4 (16.7)	0	0	0	0	11 (45.8)	1 (4.2)	7 (29.2)	7 (29.2)	4 (16.7)	1 (4.2)	1 (4.2)	14 (58.3)	3 (12.5)	8 (33.3)
	Umbilicus	0	0	0	0	0	0	0	0	0	0	0	0	0	0	0	0	1 (100)	0	1 (100)
	Uterus	0	0	0	0	0	0	0	0	0	0	0	0	0	0	0	0	3 (75)	0	0
	Vagina	0	0	2 (100)	0	0	0	0	0	0	0	0	2 (100)	2 (100)	2 (100)	0	0	0	0	0
	Wound	0	0	1 (20)	0	0	0	0	0	1 (20)	0	0	3 (60)	3 (60)	0	0	0	4 (80)	0	0
	Total	1 (1.2)	3 (3.6)	7 (8.4)	5 (6)	8 (9.6)	1 (1.2)	1 (1.2)	1 (1.2)	2 (2.4)	26 (31.3)	1 (1.2)	17 (20.5)	18 (21.7)	12 (14.5)	1 (1.2)	13 (15.7)	44 (53)	6 (7.2)	21 (25.3)

**TABLE 3:** Distribution (%) of resistance to antimicrobial classes in the genomes of *Trueperella pyogenes* from different host species and anatomical body sites (*n* = 83)

		Aminoglycosides	Biocides	Glycopeptides	MLSb	Phenicols	Sulfonamides	Tetracyclines
Host species	Bison	0	0	0	0	0	0	0
	Cattle	12 (20.7)	15 (25.9)	18 (31)	19 (32.8)	1 (1.7)	15 (25.9)	42 (72.4)
	Water buffalo	0	0	0	0	0	0	1 (50)
	Dog	0	0	0	0	0	1 (100)	0
	Goat	0	0	0	1 (100)	0	0	0
	White-tailed deer	1 (33.3)	1 (33.3)	0	0	0	1 (33.3)	2 (66.7)
	Chamois	0	0	0	0	0	0	0
	Horse	0	0	0	0	0	0	1 (100)
	Cat	0	0	0	0	0	0	0
	Sheep	0	0	3 (100)	1 (33.3)	0	0	3 (100)
	Swine	2 (18.2)	1 (9.1)	0	5 (45.5)	2 (18.2)	1 (9.1)	9 (81.8)
Anatomical body site	Abscess	0	0	0	0	0	0	1 (100)
	Foot	0	0	0	0	0	0	1 (100)
	Joint	0	0	0	0	0	0	0
	Liver	0	0	1 (100)	1 (100)	0	0	1 (100)
	Liver abscess	0	0	2 (66.7)	2 (66.7)	0	0	3 (100)
	Lung	5 (16.1)	5 (16.1)	8 (25.8)	11 (35.5)	2 (6.5)	5 (16.1)	22 (71)
	Mammary	0	0	1 (100)	0	0	0	1 (100)
	Milk	0	0	0	0	0	0	1 (100)
	N/A	0	0	0	1 (50)	0	1 (50)	2 (100)
	Nasal swab	0	0	0	0	0	0	0
	Peri fluid	0	0	0	0	0	0	1 (100)
	Rumen tissue	7 (29.2)	7 (29.2)	8 (33.3)	11 (45.8)	0	7 (29.2)	15 (62.5)
	Umbilicus	0	0	1 (100)	0	0	0	1 (100)
	Uterus	0	0	0	0	0	0	3 (75)
	Vagina	2 (100)	2 (100)	0	0	0	2 (100)	2 (100)
	Wound	1 (20)	3 (60)	0	0	1 (20)	3 (60)	4 (80)
	Total	15 (18.1)	17 (20.5)	21 (25.3)	26 (31.3)	3 (3.6)	18 (21.7)	58 (69.9)

### Phenotypic antimicrobial susceptibility

**Fig 5:**
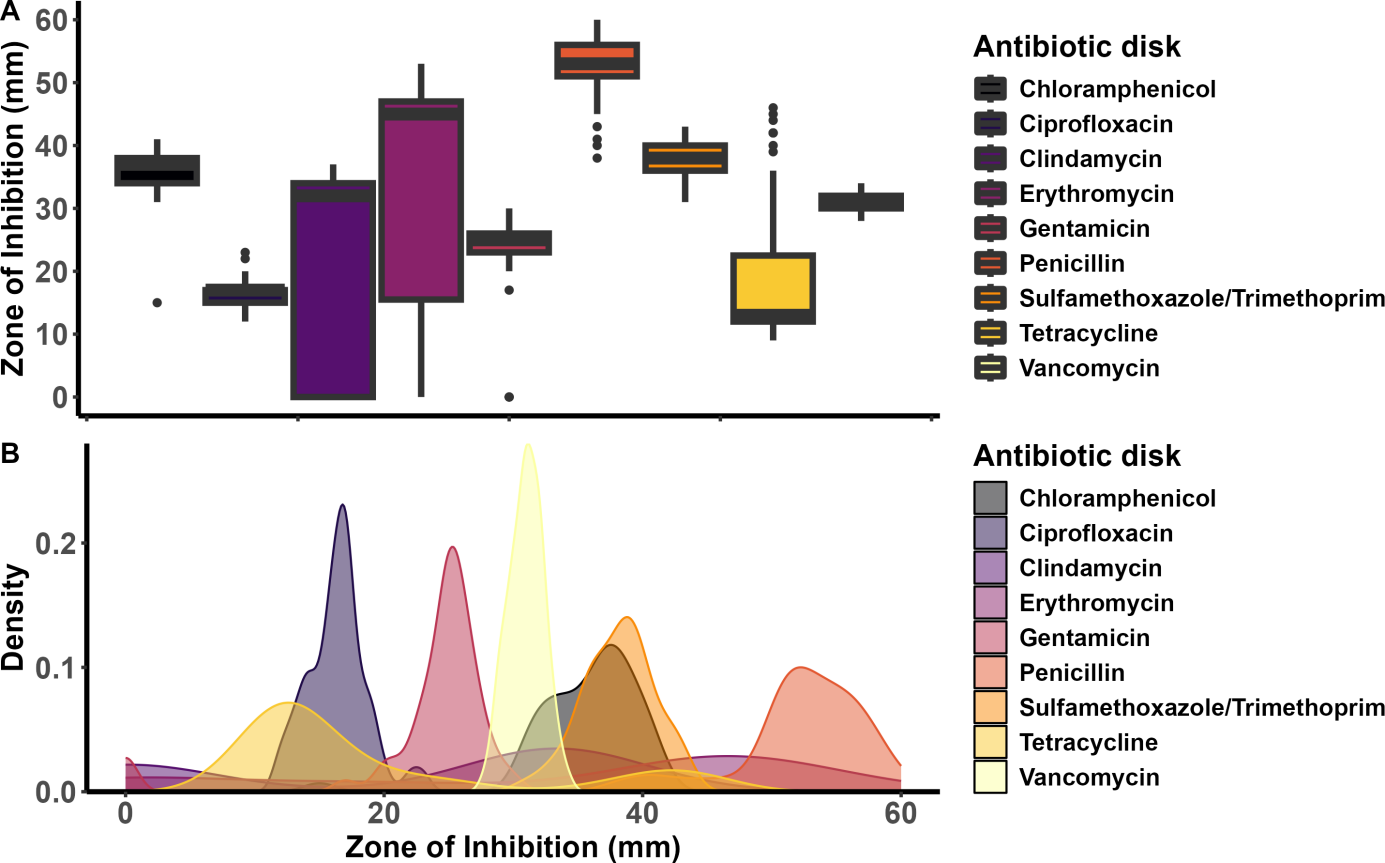
Differences in the (**A**) variation and (**B**) density of ZOI diameters (mm) for each tested antibiotic across selected *Trueperella pyogenes* isolates (*n* = 49).

The zone of inhibition (ZOI; in mm) produced by each of the antibiotic disks tested ranged from 0 to 37 (21.1 ± 2.3 [SEM]) for clindamycin, 0–53 (32.2 ± 2.8) for erythromycin, 15–41 (35.8 ± 0.6) for chloramphenicol, 12–23 (16.4 ± 0.3) for ciprofloxacin, 0–30 (23.4 ± 0.9) for gentamicin, 38–60 (52.3 ± 0.7) for penicillin, 31–43 (38.0 ± 0.4) for sulfamethoxazole/trimethoprim, 9–46 (20.1 ± 1.7) for tetracycline, and 28–34 (21.1 ± 0.2) for vancomycin (Table S4). The ZOI was the largest for penicillin, followed by erythromycin, sulfamethoxazole/trimethoprim, chloramphenicol, and vancomycin. Certain isolates (i.e., isolates 2022_1_96 and 2022_1_29 from cattle ruminal tissue, and 23_50711 from cattle lungs) were also completely resistant (0 mm ZOI) to gentamicin, clindamycin, or erythromycin (Fig. 5).

**Fig 6:**
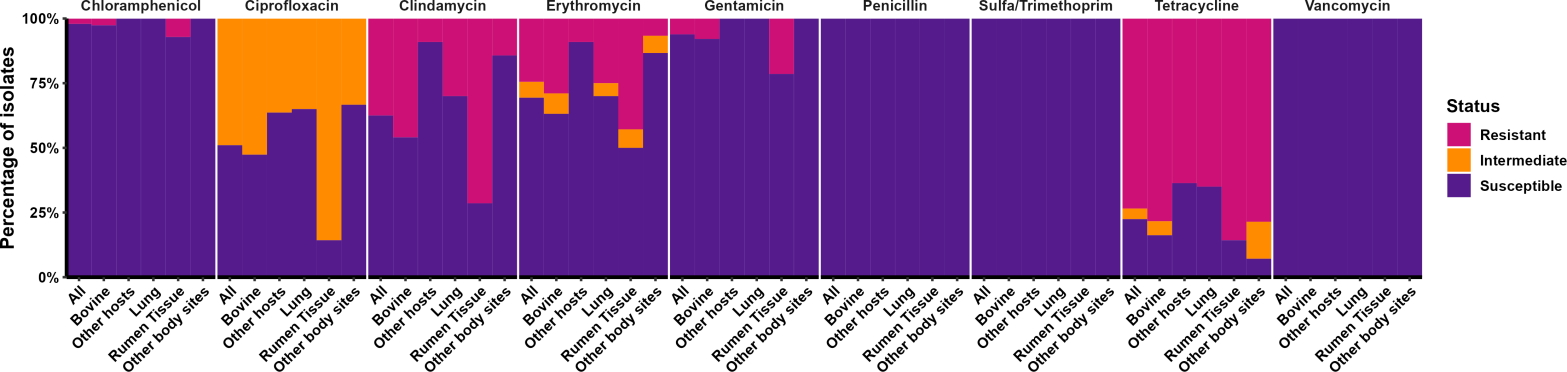
The proportion of resistant, intermediate, and susceptible *Trueperella pyogenes* isolates originating from different animal hosts and body sites based on disk diffusion (*n* = 49).

According to the breakpoints available for *Streptococcus pneumoniae*, the majority of *T. pyogenes* isolates were resistant to tetracycline (75%), followed by clindamycin (37.5%), erythromycin (24.5%), gentamicin (6.1%), and chloramphenicol (2.1%) (Table 4). None of the *T. pyogenes* isolates tested were resistant to ciprofloxacin, sulfamethoxazole/trimethoprim, penicillin, or vancomycin. Concomitantly, 49% of isolates had intermediate resistance to ciprofloxacin, 6.1% to erythromycin, and 4.2% to tetracycline (Table 4). When comparing different animal hosts and body sites, cattle isolates were more frequently resistant to tetracycline, erythromycin, clindamycin, ciprofloxacin, and chloramphenicol than the other animal hosts combined. Additionally, *T. pyogenes* isolates from the ruminal tissue were mostly resistant to erythromycin, clindamycin, ciprofloxacin, and chloramphenicol than the lung and all the other body sites-associated isolates (Fig. 6).

**TABLE 4:** Disk diffusion zone of inhibition (ZOI) and phenotypical antibiotic resistance *Trueperella pyogenes* isolates against selected antibiotics (*n* = 49)^*a*^^,^^*e*^

	Zone diameter (mm)									Phenotype		
Antibiotic disk	0–5	6-10	11-15	16–20	21–25	26–30	31–35	36–40	>40	S	I	R
Gentamicin^*d*^	6.10%	-	-	6.10%	51.00%	36.70%	-	-	-	46 (93.9%)	0	3 (6.1%)
Ciprofloxacin^*b*^	-	-	30.60%	65.30%	4.10%	-	-	-	-	25 (51%)	24 (49%)	0
Erythromycin^*c*^	20.40%	2.00%	2.00%	6.10%	8.20%	-	-	-	61.20%	34 (69.4%)	3 (6.1%)	12 (24.5%)
Sulfamethoxazole/trimethoprim^*c*^	-	-	-	-	-	-	16.30%	67.30%	16.30%	49 (100%)	0	0
Clindamycin^*c*^	35.40%	2.10%	-	-	-	4.20%	47.90%	10.40%	-	30 (62.5%)	0	18 (37.5%)
Chloramphenicol^*c*^	-	-	2.10%	-	-	-	35.40%	56.30%	6.30%	47 (97.9%)	0	1 (2.1%)
Penicillin^*c*^	-	-	-	-	-	-	-	4.10%	95.90%	49 (100%)	0	0
Vancomycin^*c*^	-	-	-	-	-	34.70%	65.30%	-	-	49 (100%)	0	0
Tetracycline^*c*^	-	6.30%	52.10%	12.50%	6.30%	2.10%	-	20.80%	-	10 (20.8%)	2 (4.2%)	36 (75%)

^*a*^ Vertical red lines represent the breakpoints for the tested antibiotic disks.

^*b*^ Coryneform breakpoints.

^*c*^ *Streptococcus pneumoniae* breakpoints.

^*d*^ ZOI = 0 resistance breakpoint.

^*e*^ S, susceptible; I, intermediate; R, resistant.

### Comparison between genotypic and phenotypic antimicrobial susceptibility

**Fig 7:**
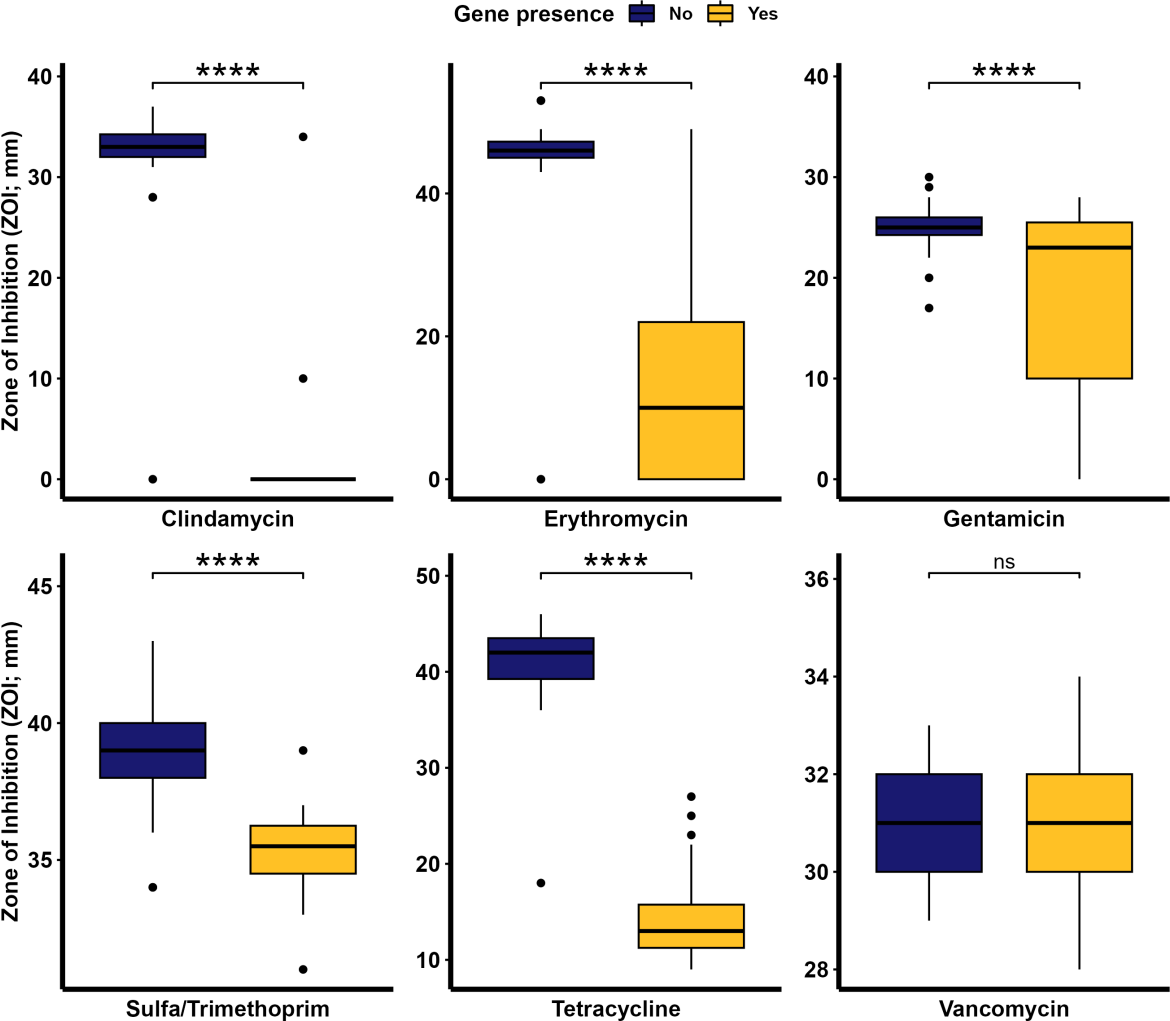
Disk diffusion zone of inhibition (mm) for antimicrobials against *Trueperella pyogenes* isolates (*n* = 49) based on the presence or absence of corresponding antimicrobial resistance genes in their genomes. **** indicates a significant difference with *P* value < 0.001 and ns indicates no significance (*P* value > 0.05). Of note, the diameter of the disk was 6 mm.

We sought to identify whether and to what extent the presence of an ARG influenced phenotypic AMR in *T. pyogenes*. To address this, differences in the ZOI between strains with or without corresponding ARG(s), were determined with the Kruskal-Wallis test. The presence of ARGs in the isolates resulted in a significantly smaller ZOI (Fig. 7). Isolates that carried ARGs conferring resistance to clindamycin, erythromycin, gentamicin, sulfamethoxazole/trimethoprim, or tetracycline had a significantly (*P* < 0.05) smaller ZOI than those isolates that lacked the ARGs. The mean ZOI for vancomycin was similar (*P* = 0.49) between isolates that had *vanG* versus the ones that did not (Fig. 7), again demonstrating that *vanG* provides only low-level resistance to vancomycin. Additionally, the correlation between genotypic and phenotypic AMR was assessed using either the chi-square or Fisher’s exact test. There was a positive correlation (*P* < 0.05) between genotypic and phenotypic resistance for aminoglycosides, macrolides, tetracyclines, sulfonamides, and lincosamides (Fig. 7), meaning that if an isolate was predicted to be resistant based on its genotype, then it was likely phenotypically resistant as well. Generally, *T. pyogenes* isolates are less susceptible to tetracycline and macrolides, while they are mostly susceptible to penicillin, vancomycin, sulfamethoxazole/trimethoprim, and gentamycin.

### Metabolic characterization of selected *T. pyogenes*

**Fig 8:**
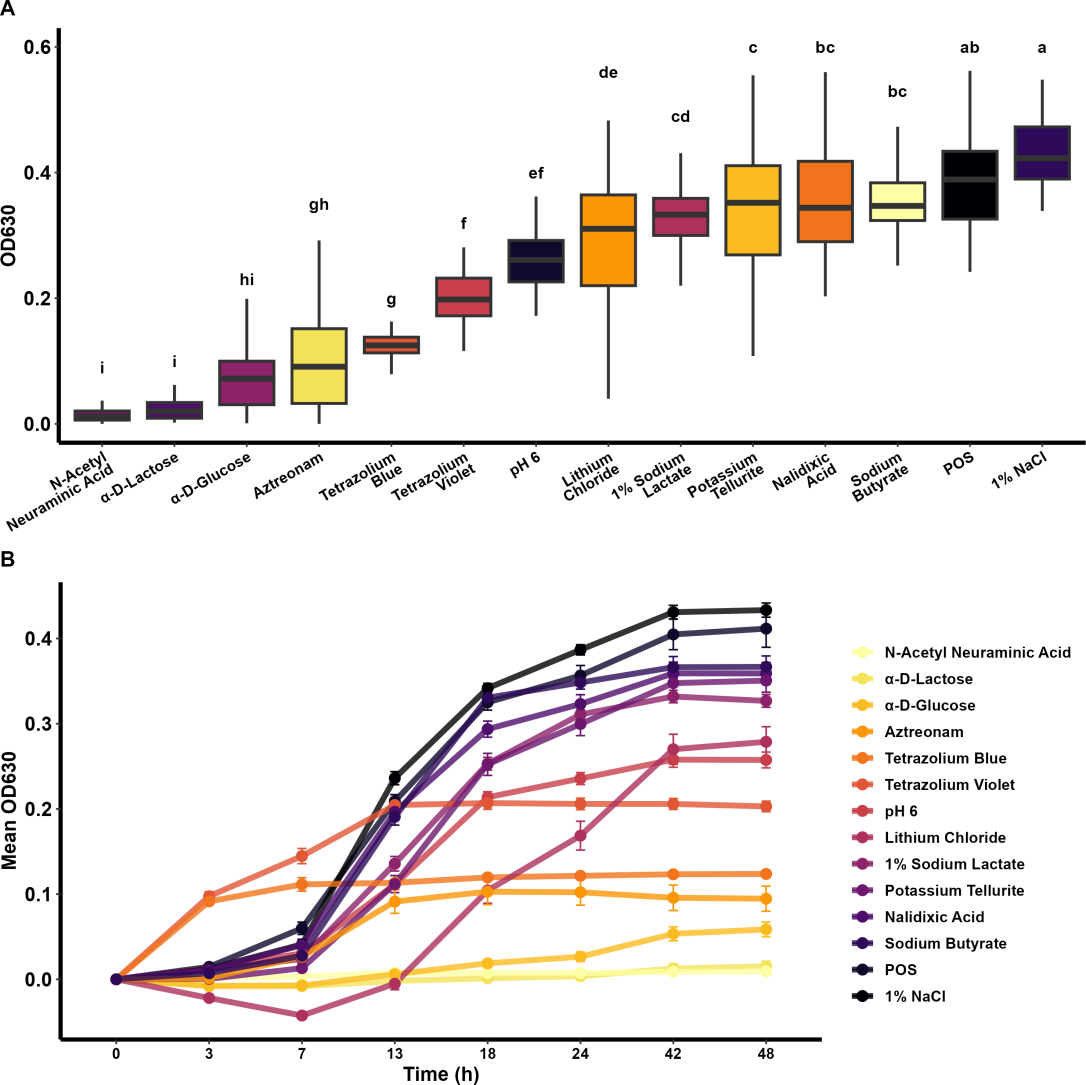
Metabolic characterization of *Trueperella pyogenes* isolates showing (**A**) the preferred substrates in average utilized in order from least to most metabolic activity, and (**B**) their metabolization over time measured by the color change in the Gen III Microplate (*n* = 49). Different letters indicate different mean OD values (*α* = 0.05). POS = positive control well.

**Fig 9:**
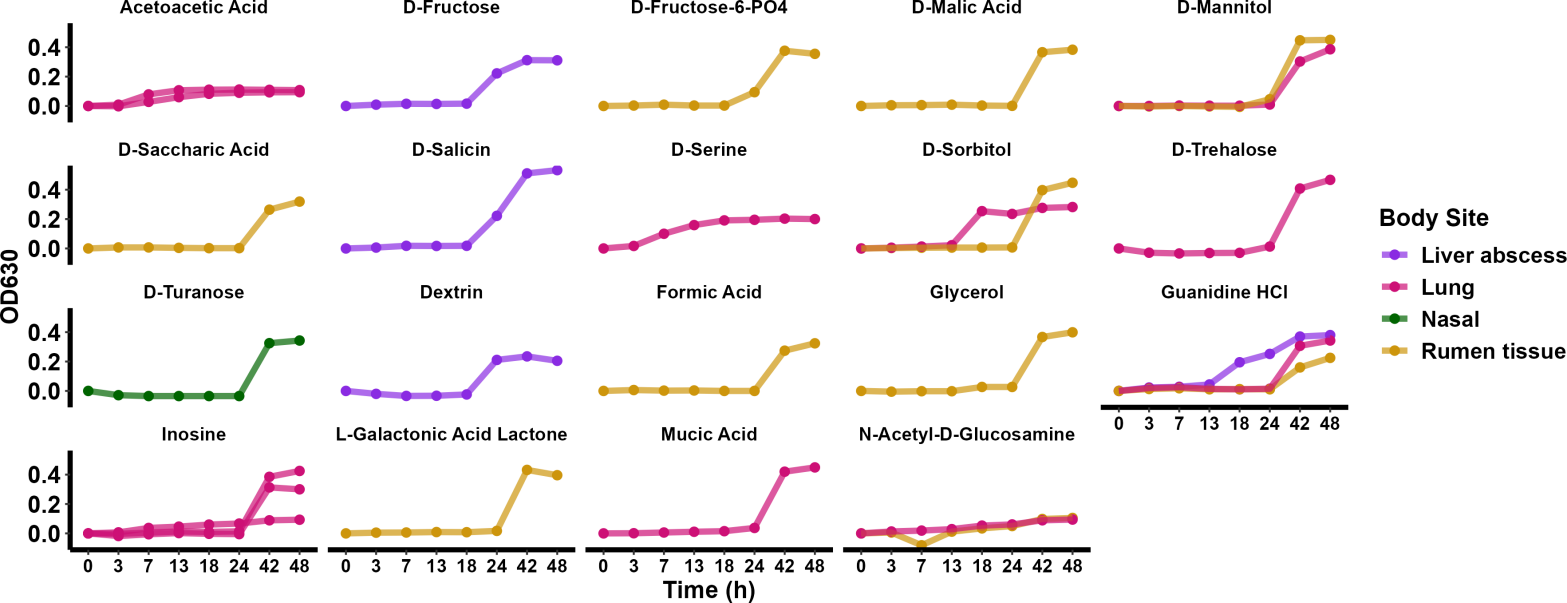
The growth curve of *Trueperella pyogenes* isolates with a longer lag phase based on the metabolism of different substrates.

To identify whether there is a common metabolic signature among *T. pyogenes* strains originating from different body sites within an animal, and from different animal hosts, we used Biology GENIII MicroPlates to biochemically profile 49 *T. pyogenes* isolates. Each strain was tested in a 96-well Microplate containing 71 carbon sources and 23 chemical sensitivity analyses (Table S5). After 48 h of incubation, all *T. pyogenes* isolates tested turned purple in the positive control, and no color change was observed in the negative control well based on the OD_630_. Most isolates grew well at a pH of 6.0 and in a low salt concentration (up to 4% NaCl) but not under acidic conditions (pH = 5) and high salt concentrations (8% NaCl). The carbon sources readily utilized by all or most of the isolates were α-d-lactose, N-acetyl neuraminic acid, and α-d-glucose (Fig. 8). Conversely, some carbon sources were not preferred by most isolates, but were metabolized by one to three isolates after a longer lag phase (Fig. 9). Most or all isolates grew normally and were able to metabolize 1% sodium lactate, tetrazolium violet, tetrazolium blue, nalidixic acid, lithium chloride, potassium telluride, aztreonam, and sodium butyrate. No specific differences in growth with various carbon sources or chemicals were observed between isolates from different animal hosts or body sites on their growth with various carbon sources or chemicals were observed in this biochemical microplate panel.

## DISCUSSION

*T. pyogenes* is an opportunistic pathogen causing suppurative infections, such as mastitis, metritis, pneumonia, and abscesses in livestock, pets, and wildlife, leading to significant health and economic losses. Zoonotic infections of *T. pyogenes* have also been reported in humans (15, 17, 18, 33, 34). Although including human isolates would help to investigate the link between animal and human infections, genomic data for human isolates were not available at the time this study was conducted. Virulence factors and AMR may contribute to pathogenesis in multiple host species and tissues and increase the risk of ARG dissemination and treatment failure. In the present study, we evaluated the genomes, pangenome, virulence genes, and ARGs of *T. pyogenes* isolated from diverse animal hosts (11 different hosts) comprised of livestock, companion, and wild animals, and from 16 different body sites.

A phylogenetic tree of 83 *T. pyogenes* genomes revealed that the cattle and swine isolates largely clustered into separate clades with the exception of a few cattle isolates that were closely related to the swine isolates. However, we did not observe any grouping based on the type of infection or body site between or within animal species. Our analysis revealed that *T. pyogenes* has an open pangenome, similar to what was observed in a previous pangenome analysis of 20 *T. pyogenes* genomes (35). Although other studies have characterized *T. pyogenes* isolated in China, India, and Europe (35–37), this is the first genomic characterization of clinical *T. pyogenes* isolates from North America.

The predominant virulence factor genotype was *plo*, *fimA*, *fimC*, *fimE*, and *nanP* (genotype VIII) and *plo*, *fimA*, *fimE*, and *nanP* (genotype XVI) which were both found in 14.5% of isolates. All but one *T. pyogenes* genome carried the *plo* gene, and 97.6% of strains from this study also encoded *fimA*. There is great variation in virulence gene distribution among studies evaluating *T. pyogenes*, with some harboring all known virulence genes as the predominant genotype (38), highlighting the versatility of this bacteria as an opportunistic pathogen. Other studies have also identified the *plo* and *fimA* genes in all *T. pyogenes* isolates from bovine mastitis and metritis samples (39), similar to our cattle strains (100% carried *plo* and *fimA*). In the present study, two of the seven virulence genes screened, *plo* and *fimA*, were present in nearly all isolates. This is consistent with other studies of virulence genes in *T. pyogenes* (14, 40–42). The *plo* gene encodes for a hemolytic exotoxin, the cholesterol-dependent cytolysin PLO, which belongs to the family of cholesterol-binding molecules in the host cell lipid membrane. Gram-positive pathogens that express these pore-forming toxins are associated with higher cholesterol concentrations in the target cell membranes (43). The PLO protein binds to host cell membranes, inducing the formation of oligomeric β-barrel pores, creating channels (44), and leading to cell lysis, in addition to the hemolysis, immune cell lysis, and cytokine expression by the host immune system (45). A previous study demonstrated that PLO is necessary for hemolysis as non-virulent *T. pyogenes plo* knockout mutants were not capable of hemolysis when injected into mice (21). Given that *plo* and *fimA* were present in almost all 83 *T. pyogenes* genomes analyzed, these two genes may be good targets for vaccine development.

Neuraminidases, encoded by *nanH* and *nanP* in *T. pyogenes*, are important virulence factors for bacteria that inhabit the mucous layer of organs and nutrient-limited environments (14). These enzymes remove sialic acid from polysaccharides, glycolipids, and glycoproteins, which in turn are used as a carbon source by the bacterium, facilitating tissue colonization by reducing the mucous layer viscosity (46–48). Neuraminidases are important in *T. pyogenes* cell adhesion (22) as they are as important as collagen-binding proteins encoded by *cbpA* (49) and fimbriae (50, 51) in cell adhesion. The high prevalence of the *fimC* and *fimE* genes among *T. pyogenes* isolates recovered from clinical mastitis cases (52) suggests that they may be important in mammary gland colonization. The risk of *T. pyogenes* mastitis is higher during early lactation and increases with recurrent infections (53). In another study, the prevalence of *cbpA* in *T. pyogenes* differed between animal hosts, where 100% of reptiles, 25% of companion animals, 54% of pig, 7% of cattle, and 0% of horse, rabbit, and rat isolates carried the gene (36). However, previous studies have failed to find a consistent association between the presence of virulence genes and animal host or infection site (14, 36, 38, 41).

*T. pyogenes* is a primary pathogen for uterine infections, and purulent uterine disease in cows has been associated with endometrial cell sensitivity to the PLO exotoxin causing hemolysis and cytolysis and an inflammatory response from the host with an increase in the accumulation of IL-1β, IL-6, and IL-8 (54). Additionally, the severity of metritis clinical symptoms was linked to the virulence genotype of *T. pyogenes* in postpartum cows (38). This interaction with the animal host immune system can help explain the opportunistic nature of *T. pyogenes*. For instance, about 40% of cows can develop pyometra after parturition, which is linked to the loss of the epithelial cell layers that can provide protection against invasion by *T. pyogenes*, *E. coli*, and other pathogens (50). In addition to virulence factors, *T. pyogenes* can survive inside host phagocytes and has been shown to persist inside macrophages for 72 h (45). It is also capable of forming biofilms that significantly increase the ability of *T. pyogenes* to evade the host immune response, attach to epithelial cells, and survive exposure to antimicrobials (19). Biofilm formation is usually linked to the persistence of infections such as bovine mastitis caused by *Staphylococcus aureus* due to the production of biofilm-associated protein (Bap) (55). Resistance to antimicrobials as a result of biofilm formation can possibly explain why treatment for *T. pyogenes* often takes a prolonged period of time for success.

Although no single ARG was found in all genomes, the most common ARGs in these isolates were those that confer resistance to tetracyclines, glycopeptides, and MLS_B_. Genotypic multidrug resistance (ARGs to three or more antimicrobial classes) was found in 43.3% (36 out of 83) of *T. pyogenes* isolates evaluated in the present study. Isolates that harbored the largest number of ARGs (*n* = 5–8 genes) originated from the cattle ruminal tissue (*n* = 6 isolates), and the lungs of cattle (*n* = 2), swine (*n* = 1), and white-tailed deer (*n* = 1). The isolates with no identified ARGs originated from the lungs of bovines, swine, and white-tailed deer, as well as the nasal cavity of a cat, and cattle ruminal tissue samples. *T. pyogenes* isolates from this study had ARGs predominantly conferring resistance to tetracyclines, followed by MLS_B_, and glycopeptides. This observation is consistent with other studies evaluating the genotypic AMR profiles of *T. pyogenes*. For example, Werckenthin et al. (56) observed a high prevalence of resistance to tetracyclines and sulfonamides in cattle- and swine-associated *T. pyogenes* isolates.

Cattle ruminal tissue isolates *T. pyogene*s 2022_1_29 and 2022_1_77 both carried the same eight ARGs conferring resistance to six different antimicrobial classes and appeared to be from the same strain based on ANI (Table S2). Notably, four of these genes *sul1*, *qacEdelta1*, *aadA2*, and *ant(2″)-Ia* were co-located together on a contig that was potentially from a plasmid. The best matches (>99.5% identity and 90% coverage) for this contig in the NCBI nucleotide database were to species in the *Enterobacterales* order such as *E. coli* (CP050163.1)*, Citrobacter freundii* (CP038659.1)*, Proteus mirabilis* (CP137083.1), and *Salmonella enterica* subsp. *enterica* (OX442404.1). This same contig was also identified in *T. pyogenes* 2022_1_75. A different cattle ruminal tissue-associated isolate, 2023_3_67, also encoded eight ARGs conferring resistance to five different antimicrobial classes. In this isolate, the *erm*(X) and *tet*(33) genes were co-located on an 8,048 bp contig that was nearly identical (99.95% identity and 100% coverage) to a plasmid (pAP2; AY255627.1) in *T. pyogenes* (57). The *erm*(X) and *tet*(33) genes were also found on a plasmid-like contig in isolates 2022_1_30 and 2022_1_96; however, in strains 2022_1_27 and 306 they appeared to be chromosomally encoded as they were located on contigs that were approximately 1.3 Mbp in length (data not shown). Some infections that have *T. pyogenes* as one of the primary causative agents, such as endometritis in dairy cows, are treated with antimicrobials. If AMR increases among *T. pyogenes* or spreads between *T. pyogenes* and the other bacterial species through horizontal gene transfer (HGT) this can lead to a reduction in treatment efficacy and economic losses due to lower milk yields and decreased fertility in the herd. Choosing the correct antimicrobial based on the intrinsic and acquired AMR of *T. pyogenes* is therefore important.

HGT in the gut can spread ARGs among commensal and pathogenic bacteria (58, 59). The spread of AMR within the microbiomes of animals as well as farm workers has been demonstrated through HGT via plasmids. This includes tetracycline-supplemented feed given to poultry (60), nalidixic acid in cattle and swine (61), tetracycline and sulfonamide resistance gene-carrying plasmids in cattle (62), and methicillin-resistant *S. aureus* from farm animals (63). An increase in the demand for food/meat and the consequent intensification of farming practices for livestock are associated with the increase in the genotypic and phenotypic prevalence of AMR in animals (64). This has the potential to also impact AMR in humans (65, 66). We also see a trend of increased AMR in chickens, pigs, and cattle observed in mid- and lower-income countries for the past two decades (67). Other studies have observed that *T. pyogenes* isolates are genetically close to bovine isolates (68).

Unfortunately, there are no established breakpoints for *T. pyogenes* based on the disk diffusion assay. Therefore, resistance breakpoints for *S. pneumoniae* and *Corynebacterium* spp. and related coryneform bacteria were used in the present study. As such, caution is warranted when extrapolating our results to clinical settings. The *in vitro* disk diffusion assay demonstrated that *T. pyogenes* isolates from the present study were predominantly resistant to tetracycline (75%), clindamycin (37.5%), and erythromycin (24.5%), with a smaller percentage resistant to gentamicin (6.1%) and chloramphenicol (2.1%). Conversely, our isolates were completely susceptible to penicillin, sulfamethoxazole/trimethoprim, and vancomycin. This is in accordance with other studies, where resistance to tetracycline, as high as 85.5% of tested isolates (52), and macrolides, (around 30–35% of isolates) (38, 40, 69), are predominant.

Differences in the *in vitro* antimicrobial susceptibility of *T. pyogenes* can be observed depending on the antimicrobial used, sample origin, and animal species. For instance, *T. pyogenes* of bovine origin exhibited resistance to tetracyclines that differed based on the tetracycline (6% doxycycline, 21% tetracycline, and 88% oxytetracycline) (31), and infection site (11% in metritis vs. 35% in mastitis) (38, 40). *T. pyogenes* antimicrobial susceptibility can also differ based on host species as cattle and swine isolates often show diminished susceptibility to antimicrobials used in animals (e.g., tetracyclines, sulfonamides, and clindamycin) compared to isolates from other livestock and wildlife species (32, 37, 70). This is likely due to the greater direct exposure to antimicrobials in livestock than their wildlife counterparts.

Some antimicrobials, such as tetracyclines and macrolides, have traditionally been used at subtherapeutic doses in cattle and swine production systems to promote growth and prevent disease (71). This practice has likely resulted in the frequent exposure of *T. pyogenes* associated with food-producing animals to non-lethal,selective concentrations of these antimicrobials. *T. pyogenes* infections often involve multiple bacterial species and, consequently, it is not usually the main target for antimicrobial treatment. However, beta-lactams (penicillins, cephapirin, and ceftiofur), tetracyclines, MLS_B_ (erythromycin, tylosin, clindamycin), trimethoprim-sulfamethoxazole, and enrofloxacin are typically indicated for *T. pyogenes*-related infections (5). As observed in the present study, as well as with cattle in France (72) and Switzerland (68), and swine in China (73), *T. pyogenes* is widely susceptible to beta-lactams, compared to tetracyclines and MLS_B_. The most effective antimicrobials against *T. pyogenes* isolates tested in the present study were ciprofloxacin, sulfamethoxazole/trimethoprim, penicillin, and vancomycin. Known beta-lactamase genes conferring resistance to beta-lactams were not found in any of the *T. pyogenes* isolates and they were all classified as susceptible based on their phenotype. Comparisons between studies are limited due to differences in methods (microbroth vs. disk diffusion) and the breakpoints used (74).

Some isolates had reduced susceptibility to certain antimicrobials even when no known ARGs for that antimicrobial or antimicrobial class were identified, suggesting that resistance may have been due to a novel antimicrobial gene or other mechanism. It has previously been demonstrated that factors such as silent (unexpressed) genes, low copy numbers, and/or a large distance from the promoter region, can lead to a mismatch between the genotypic and phenotypic antimicrobial susceptibility profiles (75–77). The expression of phenotypic resistance without a corresponding ARG in the genome indicates that other mechanisms, such as multidrug resistance efflux pumps, may be involved in conferring resistance to the antibiotic (78).

The greatest difference in ZOI was observed for tetracycline, erythromycin, and clindamycin, especially between swine and cattle isolates. Cross-resistance to antimicrobials in the MLS_B_ class is expected in isolates carrying the *erm*(X) gene due to their structural similarities (79). Other mastitis pathogens are also known to carry MLS_B_ resistance genes, highlighting the need for judicious use of these antimicrobials for therapeutic purposes (80). Isolates from the majority cattle clade (Fig. 1) showed greater resistance to clindamycin, erythromycin, and tetracycline when compared to isolates in the swine clade, indicating possible host-specific AMR differences. Globally, the US ranks as the third largest user of veterinary antimicrobials by volume and it is projected that this will increase by 3.8% by 2030 (81). Antimicrobials are often used in livestock for one of three goals: growth promotion, prevention/metaphylaxis, or disease treatment. The most commonly used antimicrobials in this industry are tetracyclines, penicillins, macrolides, sulfonamides, aminoglycosides, lincosamides, cephalosporins, and fluroquinolones (71). However, a more comprehensive analysis between antimicrobial usage in animal production and genotypic/phenotypic AMR is needed.

Extensive antimicrobial use and misuse in livestock may drive an increased prevalence of AMR. The presence of genetically similar strains of *T. pyogenes* in humans and cattle also indicates spillover, posing a risk for humans (82). Moreover, it is essential to consider the broader context of ARG acquisition beyond antimicrobial use in livestock. *T. pyogenes* occupies diverse niches across domestic and wild animals, and this ecological versatility may expose it to a variety of microbial communities, some of which carry ARGs that could transfer through HGT. Additionally, environmental factors such as soil and water contaminated with antimicrobial-resistant bacteria may contribute to the spread of ARGs (83). By assessing AMR both genomically and phenotypically, we aimed to identify specific resistance determinants and their potential sources, which can range from environmental reservoirs to microbial interactions in commensal niches. This approach not only clarifies the distribution of AMR but also aids in developing targeted interventions to mitigate AMR transmission across animal populations and into the broader ecosystem.

Despite contributing 59 new *T. pyogenes* genomes in the present study, there remains a shortage of publicly available genomes, especially from animal species other than cattle and pigs. Since *T. pyogenes* has been isolated from numerous domestic and wild animals, obtaining more isolates from these underrepresented hosts is essential for understanding potential host-specific differences in AMR and virulence. Such characterization is key for epidemiological and evolutionary studies, revealing the adaptability of *T. pyogenes* across diverse microbial niches and its role as both a commensal and pathogen in various host species. Determining genotypic and phenotypic AMR can also guide clinical antimicrobial therapy and support efforts to prevent the development and horizontal spread of ARGs among animal and zoonotic pathogens, which is crucial for public health and the welfare of livestock.

The preferred carbon source utilization, as well as pH and salinity tolerance, were similar among all tested *T. pyogenes* isolates (Fig. 9). The OD_630_ was higher for isolates grown in 1% NaCl, suggesting that this osmotic concentration is favorable to the growth of *T. pyogenes*. Similarly, the growth of lactic acid-producing bacteria is enhanced by the addition of 1% NaCl to the medium (84). *T. pyogenes* isolates generally metabolize sodium butyrate, a short-chain fatty acid known for its pro-apoptotic effects (85). Derived from glucose metabolism, sodium butyrate serves as a nutrient source that supports bacterial growth and potentially influences host-pathogen interactions. It also has bactericidal effects against Gram-negative bacteria by inhibiting DNA synthesis with its optimal bactericidal activities occurring at low concentrations (85). Furthermore, *T. pyogenes* grows in the presence of potassium tellurite, which is used in media selective for *Corynebacterium* spp. This compound is toxic to many bacteria but is inherently tolerated by certain Gram-positive microorganisms, such as *Corynebacterium diphtheriae* and *S. aureus* (86, 87). This resistance extends beyond potassium telluride to encompass other antimicrobials and oxidative stressors, conferring a competitive advantage in hostile environments. Additionally, *T. pyogenes* metabolizes 1% sodium lactate, a commonly used food preservative due to its pH-lowering and water activity-reducing properties (88).

The ability of *T. pyogenes* isolates to degrade lithium chloride, although rare, suggests a broader spectrum of metabolic versatility that is potentially mediated by specific genetic mechanisms such as cation transporters. This underscores its adaptability to nutrient-depleted environments, where it can utilize alternative carbon sources to sustain growth and survival. Conversely, there were some exceptions to the core metabolizable carbon sources and resistance to chemical compounds. A few isolates (Table S5) demonstrated the ability to metabolize diverse carbon sources and grow in the presence of chemical compounds that the majority of *T. pyogenes* isolates could not. This metabolic flexibility may contribute to their survival, offer a competitive advantage, and may influence their pathogenicity. One advantage of this is their ability to utilize complex carbohydrates like d-trehalose and d-fructose-6-PO_4_, which serve as an energy source and protect against environmental stressors such as osmotic stress and freezing (89).

The one *T. pyogenes* isolate that was capable of metabolizing d-fructose-phosphate as a sole carbon source came from the ruminal tissue of cattle. Fructose-6-phosphate is broken down by the phosphoketolase enzyme during carbohydrate metabolism (90). Commensal ruminal bacteria, such as *T. pyogenes* and *Fusobacterium necrophorum*, can travel to the liver and cause abscesses, despite the bactericidal effects of bile in that environment. Certain species such as *F. necrophorum* and *Bifidobacterium* spp. that show resistance to bile salts also had high phosphoketolase activity when compared to their susceptible counterparts, suggesting that resistance to bile is an advantageous adaptation (90). Moreover, metabolic pathways involve the use of organic acids, such as l-galactonic acid, d-malic acid, mucic acid, saccharic acid, acetoacetic acid, and formic acid, which have bactericidal properties and change the activities of various metabolic pathways (91, 92). A ruminal tissue *T. pyogenes* isolate metabolized formic acid, which is a one-carbon product of ruminal acetate fermentation and represents about 5% of volatile fatty acids in the rumen (93). By metabolizing these organic acids, *T. pyogenes* may overcome environmental pressure and gain a competitive edge in hostile environments, such as the rumen, where formic acid is abundant.

Furthermore, *T. pyogenes* utilized sugars like d-sorbitol and d-mannitol, which are prevalent in plant tissues and host environments. d-mannitol is metabolized by the mannitol-1-phosphate dehydrogenase (M1PDH) enzyme in *S. aureus* (94)*,* contributing to pH and osmotic tolerance (95, 96). The mannitol pathway enables some microorganisms to overcome environmental pressures by reversibly transforming mannitol-1-phosphate (M1P) into fructose-6-phosphate (F6P) with M1PDH. Mannitol is further converted to fructose by the mannitol-2-dehydrogenase (M2DH) enzyme in bacteria such as *S. aureus*, *Clostridium* spp., *Bacillus* spp., *E. coli*, and *Klebsiella pneumoniae* (94, 97).

Other carbon sources utilized by a few *T. pyogenes* isolates included d-trehalose, a disaccharide produced by algae and plants. Its metabolism can be associated with abiotic stress as it protects against osmotic stress and freezing (98, 99). A few d-trehalose formation/degradation pathways are present in bacteria (100) and metabolism of this compound has been linked to pathogenicity (101). The cattle *T. pyogenes* isolate that was able to use d-trehalose as a sole carbon source came from a lung infection. Pathogenic bacteria can utilize mannitol as a carbon source. In addition to carbon sources, *T. pyogenes* has the capacity to metabolize amino acids such as d-serine and nucleosides like inosine. Interestingly, one *T. pyogenes* isolate from ruminal tissue was able to metabolize glycerol. Glycerol can enter the bacterial cell through the glycerol facilitator GlpF protein and is converted into glycerol-3-phophate by the action of the glycerol kinase (GlpK) and glycerol dehydrogenase enzymes in the cytoplasm (102, 103). Other bacterial species that can grow with glycerol as a sole carbon source include *Citrobacter freundii*, *K. pneumoniae*, *Clostridium* spp., *Enterobacter* spp., and *Lactobacillus* spp. (104–109).

The concentration and availability of carbon sources and amino acids vary depending on the site within the host (110, 111). Nutrient availability and bacterial adaptation to it play a role in niche-specificity (112). Bacteria that can infect and cause disease in diverse host body sites typically carry virulence genes. The expression of these genes is influenced by the environmental conditions, as observed in different pathotypes of *E. coli* (112) and virulence capsule expression in *S. pneumoniae* (113). Bacteria face many challenges during host colonization, particularly from the environmental conditions. Commensal and pathogenic bacteria that adapt to these conditions are best able to survive. This metabolic versatility is crucial for understanding the ecology of *T. pyogenes* and its ability to thrive amidst the challenges posed by varying host environments.

Future research should include a broader range of isolates from diverse animal species and anatomical sites to create a comprehensive database for *T. pyogenes*. This would allow for better identification of the factors involved in the development of infection and pathogenesis. In addition, long-read sequencing would enable the full assembly of putative plasmid sequences. The presence of highly conserved genes, like the virulence gene *plo*, could be used as a target for vaccine or therapeutic development. Since *T. pyogenes* is often involved in polymicrobial infections, *in vitro* studies alone may not fully capture its *in vivo* pathogenicity and virulence, especially when there are interactions with other bacteria. For example, the formation of liver abscesses in conjunction with *F. necrophorum* and *Salmonella* spp., or interactions with *S. aureus*, *Streptococcus agalactiae*, and *E. coli* in mastitis pathogenesis, highlights the complexity of these infections. Models involving multiple pathogens need to be tested to evaluate the synergistic effects of metabolite interactions between these pathogens and their resulting impact on virulence, pathogenicity, and adaptation for infection (114). Finally, the zoonotic potential of *T. pyogenes* should not be overlooked and warrants further investigation.

### Conclusions

Genomic characterization of *T. pyogenes* from various animal hosts and anatomical body sites revealed some clustering of cattle and swine isolates, with no clear distinction for other host species or body sites. Antimicrobial susceptibility testing showed that *T. pyogenes* isolates were least susceptible to tetracycline, clindamycin, and erythromycin. Cattle isolates displayed decreased susceptibility to tetracycline, erythromycin, clindamycin, ciprofloxacin, and chloramphenicol compared to isolates from other animal hosts. These findings suggest that *T. pyogenes* strains are not specific to a particular host or body site. Overall, the metabolic flexibility and tolerance of *T. pyogenes* to environmental stress, coupled with its ability to utilize a range of carbon sources and withstand toxic compounds, underscore its adaptive strategies for survival and pathogenicity. These attributes highlight the bacterium’s ability to thrive in diverse and challenging environments, contributing to its role as a significant pathogen in several host species.

## MATERIALS AND METHODS

### Isolate collection and culturing conditions

A total of 60 *T. pyogenes* isolates were recovered from six different animal hosts and 11 body sites (Table S1) and subjected to whole-genome sequencing. These isolates were from animals originating from the Midwestern United States and isolated by three different labs (*n* = 27, Diagnostic Medicine/Pathobiology, Kansas State University, Manhattan, KS, USA; *n* = 30, Veterinary Diagnostic Laboratory [VDL], North Dakota State University [NDSU]; and *n* = 2, Department of Microbiological Sciences, NDSU). Isolates from Kansas State University were isolated using sheep blood agar (Remel, Thermo Fisher Scientific Inc., Lenexa, KS, USA) incubated in a 5% CO_2_ incubator and then stored in brain heart infusion broth (BHI; BD, Franklin Lakes, NJ, USA) with 20% glycerol at −80°C before being shipped to our lab at NDSU. All *T. pyogenes* strains isolated by the VDL were clinical isolates from samples submitted for diagnosis. The remaining *T. pyogenes* strains (93CBB and 51CBC) were isolated in our laboratory from vaginal swabs taken from healthy beef cattle that were housed and raised at NDSU. In addition, *T. pyogenes* strain NCTC 5224 (ATCC-19411, American Type Culture Collection, Manassas, VA, USA), of swine origin, was included as a reference strain. *T. pyogenes* was isolated on tryptic soy agar supplemented with 5% defibrinated sheep’s blood (TSAb) (BD, Franklin Lakes, NJ, USA) incubated at 37°C with 5% CO_2_ for 24–48 h. Colonies were presumptively identified as *T. pyogenes* based on phenotypic characteristics and confirmed with matrix-assisted laser desorption/ionization time-of-flight (MALDI-TOF) (115). Confirmed *T. pyogenes* isolates were cryopreserved in BHI broth with 20% glycerol and stored at −80°C. For genomic DNA extraction and antimicrobial susceptibility testing, cryopreserved bacterial glycerol stocks were re-streaked onto TSAb plates and incubated at 37°C with 5% CO_2_ for 24–48 h.

### Genomic DNA extraction, library preparation, and whole-genome sequencing

Isolates (*n* = 60) were cultured on TSAb overnight at 37°C in 5% CO_2_, and a single colony was then selected, re-streaked onto TSAb and cultured overnight at 37°C in 5% CO_2_ to ensure the purity of the bacterial colony. One colony was used to inoculate 5 mL of BHI broth incubated overnight at 37°C in 5% CO_2_. About 1 mL of overnight culture was subjected to genomic DNA extraction using a DNeasy Blood and Tissue kit (Qiagen, Valencia, CA, USA) with an enzymatic lysis pre-step, as described previously (116, 117). The DNA quality was determined by a NanoDrop ND-1000 spectrophotometer and the concentration was measured using the Quant-iT PicoGreen dsDNA kit with a Qubit 4 fluorometer (Thermo Fisher Scientific, Waltham, MA, USA) and then stored at −20°C until library preparation. DNA libraries were constructed using the DNBSEQ short-read library preparation protocols. Libraries were sequenced using the DNA-based nanoball technology DNBSEQ on a DNBSEQ-G50 platform (MGI Tech, Shenzhen, China) with a PE150 flow cell and 2 × 150 bp sequencing length. After sequencing, raw reads were quality filtered with sequences shorter than 150 bp and >1% N content removed as well as adapter sequences, and low-quality reads (*Q* < 20) using the SOAPnuke tool (118).

### Publicly available *T. pyogenes* genome acquisition and isolate origins

In addition to the 60 *T. pyogenes* genomes sequenced in the current study, all publicly available *T. pyogenes* genomes in the National Center for Biotechnology Information (NCBI) Genome database were downloaded (*n* = 23; 22 November 2023) and included in the genomic analyses (Table S1). Only isolate genomes (i.e., non-metagenome-assembled genomes) were included. These additional genomes were included to expand the number of animal hosts and body sites available for analysis to gain insight into the host and niche-specificity. Three *T. pyogenes* ATCC 1941 assemblies were excluded as this strain was also sequenced in the present study.

### Genome assembly and annotation

Default parameters were used for all software unless specified otherwise. Genomes were assembled using SPAdes v.3.15.5 (119) with the “isolate” flag. Assemblies were filtered with BBMap v.38.96 (https://sourceforge.net/projects/bbmap/) to include only those contigs that were at least 500 bp long. Genome assembly completeness and contamination were assessed with CheckM2 v.1.0.1 (120) and genomes with greater than 5% contamination were excluded from further analysis. The quality of the assemblies was evaluated with QUAST v.5.0.2 (121) and any assembly with over 100 contigs was discarded. To improve the quality of some of the assemblies with excessive coverage, Seqtk v.1.4 (https://github.com/lh3/seqtk) was used to subsample the number of reads in each sample to approximately 100× coverage. The completeness and contamination values along with the number and size of the contigs were used to select the best assembly for each sample. Prokka v.1.14.6 (122) was used to annotate genomes with the “genus *Trueperella*” flag and a custom *Trueperella* database, which was created by downloading the annotated *T. pyogenes* reference genome ASM61205v1 assembly (GCF_000612055.1) from the NCBI database.

### Pangenome, AMR, and virulence gene analysis

The core genome of all *T. pyogenes* assemblies (*n* = 83) was determined with Roary v.3.13.0 (https://sanger-pathogens.github.io/Roary/) (123), the genes were then aligned with MAFFT v.7.475 (124) and concatenated into a core alignment. RAxML-ng v.1.2.0 (125) was used to create a phylogenetic tree with bootstrapping with 1,000 replicates and the GTR + GAMMA substitution model. The phylogenetic tree was visualized with iTol v.6.8.1 (126). The ANI between all 83 *T. pyogenes* genomes was also determined using fastANI v.1.3.4 (127, 128). ARGs were detected in the genome assemblies with RGI v.6.0.1 and CARD v.3.2.5 (129). Genomes with more than one ARG found on the same contig were visualized with Proksee (130) and genes associated with mobile genetic elements were annotated with mobileOG-db v.1.1.3 (131). A custom database of *T. pyogenes* virulence genes was created and included *cbpA* (WP_025296864.1), *fimA* (AHU90603.1), *fimC* (AHU90433.1), *fimE* (AHU90532.1), *nanH* (AAK15462.1), *nanP* (AAK98800.1), and *plo* (WP_025295886.1). All genome assemblies were screened for these genes using DIAMOND v.2.1.8.162 (132) with a 90% identity threshold. These virulence genes were chosen because of their reported significance in the pathogenesis of *T. pyogenes*.

### Phenotypic antimicrobial susceptibility testing using disk diffusion

To evaluate phenotypic AMR, a subset of the *T. pyogenes* isolates (*n* = 49) was selected and subjected to susceptibility testing against nine different antibiotics using the Kirby-Bauer disk diffusion method (133). These antibiotics were chosen based on the ARGs identified in the genomic analysis. Commercially available dried 6 mm filter paper disks containing specific concentrations of the following antimicrobial drugs were tested: erythromycin (E-15), sulfamethoxazole-trimethoprim (SXT25), tetracycline (Te-30), penicillin (P-10), ciprofloxacin (CIP-5), clindamycin (CC-2), vancomycin (Va-30), gentamicin (GM-10), and chloramphenicol (C-30) (Hardy Diagnostics, Santa Maria, CA, USA). A direct colony suspension of a 0.5 McFarland standard was prepared using 5 mL of sterile 0.85% phosphate-buffered saline (1× PBS, Thermo Fisher Scientific, Waltham, MA, USA) and a colony from a blood agar plate incubated for 24 h at 37°C with 5% CO_2_. Inoculum turbidity was measured using a MicroScan turbidity meter (Beckman Coulter, Brea, CA, USA) and a sterile cotton swab was used to spread the inoculum on the plates. The inoculum was spread in three different directions to ensure an even distribution of the culture and left to dry on the benchtop before disks were applied to the surface of the plate. Plates used for the disk diffusion assay were 100 mm cation-adjusted Mueller-Hinton agar (Oxoid, Basingstoke, Hampshire, UK) supplemented with 5% defibrinated sheep’s blood poured to a minimum depth of 4 mm, as recommended for disk diffusion testing of fastidious organisms.

*S. pneumoniae* (ATCC 49619) was used as an assay quality control (134) and three disks were used on each agar plate. Disks were removed from their cartridge using a pair of flame-sterilized tweezers, placed onto the agar plate at least 24 mm apart from each other and lightly pressed into the agar. Plates were inverted and incubated at 37°C with 5% CO_2_ for 24 h. After 24 h of incubation, the ZOI was observed and recorded using a caliper to the nearest millimeter. Isolates were classified as resistant, intermediate, or susceptible according to the CLSI-2020 M100 Performance Standards for Antimicrobial Susceptibility breakpoints for *S. pneumoniae* (135) for chloramphenicol, clindamycin, sulfamethoxazole/trimethoprim*,* tetracycline, vancomycin, and erythromycin, and the coryneform bacteria breakpoints for ciprofloxacin, as breakpoints for *T. pyogenes* are not available and have not been validated to date. Additionally, as no disk diffusion breakpoints are available for either *S. pneumoniae,* coryneform bacteria, or *T. pyogenes*, complete resistance (ZOI = 0) was used to classify an isolate as resistant to gentamicin, while any other value for the ZOI was considered susceptible for this study.

### Evaluation of the association between genotypic and phenotypic AMR

The association between the ZOI (in mm) and the presence or absence of ARGs for each of the tested antibiotics was evaluated by the Kruskal-Wallis test as the data were not normally distributed. Normality was tested with the Shapiro-Wilk test and a significance level of *α* < 0.05 was used. Correlation between the presence of an ARG (genotypic resistance) and antimicrobial susceptibility, resulting from the disk diffusion test (phenotypic resistance) was determined by Pearson’s chi-squared test with Yates' continuity correction, and when the expected values for contingency tables were smaller than 5, then the Fisher’s exact test was used. Significance was considered at *α* < 0.05. All analysis was conducted in RStudio using R v.4.3.3.

### Metabolic profiling of *T. pyogenes* isolates

Biology GENIII MicroPlates were used to biochemically profile 49 *T. pyogenes* isolates. Each *T. pyogenes* isolate was tested in a 96-well Microplate containing 71 carbon sources and 23 chemical sensitivity analyses (Table S5). The Biolog MicroPlate results show a positive reaction by the change in color to purple due to the reduction reactions of tetrazolium violet present in the substrate. Therefore, the change in color is a result of the bacterial respiration or oxidation of the substrate, rather than cell density from bacterial growth only. It is a method for measuring the adaptation and survivability of the bacteria while exposed to a single carbon source or chemical compound. Measuring the color change over time also has the advantage of showing the differences in how fast bacteria can adapt, if needed, to utilizing a carbon source or how the metabolism is impacted by these different substrates, rather than just looking at the final color change after incubation for a positive/negative reaction.

*T. pyogenes* isolates (Table 1) were transferred from 20% glycerol BHI stocks stored at −80°C onto TSAb, incubated at 37°C with 5% CO_2_, and sub-streaked two times using the same conditions. From the TSAb plate, a sterile cotton swab was used to transfer the isolated colonies into tubes containing inoculating fluid A (IF-A; Biolog, Hayward, CA, USA) to an OD of 0.2–0.3 using a MicroScan turbidity meter (Beckman Coulter, Brea, CA, USA), according to the manufacturer’s instructions. A cell density of 95–92% T, as recommended by the manufacturer for fastidious organisms such as *T. pyogenes*, was used. About 100 mL of prepared inoculum was transferred into each of the 96-well of the GenIII plate (Biolog, Hayward, CA, USA) containing 94 biochemical tests, including 71 different carbon sources and 23 chemical sensitivity analyses. Then, the OD_630_ was measured using the Gen5 Microplate reader (BioTek, Winooski, VT, USA) before incubation (0 h) and after 3, 7, 13, 18, 24, 43, and 48 h of incubation. Differences between the mean final OD values were determined using the non-parametric Kruskal-Wallis test and multiple comparisons adjusted with the Dunn Bonferroni method at a significance level *α* = 0.05; all analyses were done using the R v.4.3.3.

## Data Availability

All genome assemblies have been deposited in NCBI’s GenBank under the BioProject PRJNA1071155.

## SUPPLEMENTAL MATERIAL

Supplemental material aem.01725-24-s0001.pdf Tables S1 to S5 and Figure S1.

ASM does not own the copyrights to Supplemental Material that may be linked to, or accessed through, an article. The authors have granted ASM a non-exclusive, world-wide license to publish the Supplemental Material files. Please contact the corresponding author directly for reuse.
