# Supplementary material for: Genomic and metabolic characterization of Trueperella pyogenes isolated from domestic and wild animals
Source: Appl Environ Microbiol. 2024 Dec 31;91(1):e01725-24. doi: 10.1128/aem.01725-24 (PMC11784230; doi:10.1128/aem.01725-24)
Supplement: Supplemental material — Tables S1 to S5 and Figure S1. [file aem.01725-24-s0001.pdf]

Table S1. Summary of genomic characteristics of *Trueperella Pyogenes* isolates (n = 60).

Isolate ID	No. of contigs	Genome size (bp)	G+C content (%)	N50 value	Average coverage (x)	No. of reads	No. of coding sequences	Completeness	Contamination	CDS	tRNA	rRNA	tmRNA	Hypothetical protein	Repeat region	CRISPR cas3
306	7	2254456	59.66	1298235	547	8226988	2010	100	0.06	2010	46	3	1	808	1	1
311	8	2263274	59.6	658229	547	8257600	2004	100	0.24	2004	46	2	1	782	2	1
315	8	2257671	59.62	1224928	126	1902225	2008	99.99	0.55	2005	47	3	1	783	1	1
1259	7	2238604	59.7	767075	127	1902225	2000	100	0.61	1999	46	3	1	791	1	1
1355	10	2256793	59.68	512686	126	1902225	1991	100	0.16	1992	46	3	1	769	3	1
1361	10	2248149	59.57	518598	550	8250106	1995	99.99	0.63	1995	47	4	1	758	1	1
1409	23	2203817	59.79	240788	562	8253908	1954	99.99	0.6	1954	46	3	1	755	1	1
1446	18	2246382	59.65	270659	550	8241344	1991	100	0.23	1991	46	2	1	779	2	1
1494	88	2456439	59.27	234168	116	1902225	2186	100	1.01	2345	47	2	0	1187	3	0
1510	15	2285083	59.46	416659	542	8254128	2014	100	0.74	2014	46	3	1	786	1	1
5861	12	2338360	59.41	551249	517	8066016	2074	99.99	0.74	2074	46	2	1	825	2	1
7762	9	2316395	59.67	514415	123	1902225	2074	100	0.36	2074	46	3	1	868	3	1
8896	14	2291294	59.44	425551	125	1902225	2021	100	0.72	2018	46	3	1	793	1	1
2022_1_14	8	2257608	59.62	1489978	545	8206002	2009	99.99	0.56	2009	47	4	1	788	1	1
2022_1_27	8	2254038	59.66	1298478	550	8263384	2011	100	0.06	2011	46	2	1	808	1	1
2022_1_29	21	2297840	59.57	333606	124	1902225	2036	100	0.66	2035	46	2	1	802	2	1
2022_1_30	25	2345986	59.59	326905	122	1902225	2072	99.99	0.96	2074	46	3	1	835	3	1
2022_1_75	12	2297887	59.83	574460	536	8213734	2041	100	0.73	2041	46	2	1	814	2	1
2022_1_77	24	2297986	59.57	271189	124	1902225	2036	100	0.65	2039	46	3	1	808	1	1
2022_1_79	8	2245238	59.68	514558	549	8219606	2003	100	0.03	2003	46	2	1	809	1	1
2022_1_94	7	2246587	59.67	733694	127	1902225	1996	100	0.29	1993	46	4	1	770	2	1
2022_1_96	20	2348067	59.54	512190	523	8180744	2070	99.99	0.61	2070	46	3	1	823	1	1
2023_3_104	9	2341381	59.58	803559	514	8020296	2115	100	0.38	2115	46	3	1	879	1	1
2023_3_111	10	2221839	59.73	740222	128	1902225	2000	99.99	0.12	2002	46	3	1	801	0	0
2023_3_119	13	2253521	59.8	382483	127	1902225	1997	100	0.15	2000	46	3	1	789	1	1
2023_3_163	7	2237593	59.7	683021	557	8309352	1995	100	0.61	1995	46	3	1	787	1	1
2023_3_164	7	2230933	59.75	527281	544	8092152	1976	99.99	0.12	1976	46	3	1	774	1	1
2023_3_167	12	2270361	59.64	753764	532	8052916	2018	100	0.74	2018	46	3	1	809	1	1
2023_3_174	6	2237639	59.7	767312	128	1902225	1995	100	0.61	1997	46	4	1	790	1	1
2023_3_175	10	2183239	59.77	1658981	131	1902225	1938	99.99	0.12	1937	46	3	1	740	0	0
2023_3_235	11	2204999	59.7	392722	560	8232162	1954	99.99	0.1	1954	46	3	1	747	1	0
2023_3_4	13	2320291	59.54	463034	123	1902225	2062	99.99	0.36	2064	46	3	1	821	2	1
2023_3_60	15	2194739	59.74	538925	130	1902225	1950	99.99	0.13	1946	46	4	1	740	0	0
2023_3_67	21	2401717	59.57	388207	501	8019418	2135	99.99	0.73	2135	46	3	1	878	1	1

2023_3_71	18	2267157	59.73	335705	126	1902225	2028	100	0.23	2027	46	3	1	819	2	1
2023_3_72	5	2234189	59.71	1705908	542	8069574	1972	100	0.05	1972	47	3	1	769	1	1
2023_4_80	15	2294512	59.53	493322	124	1902225	2040	99.99	0.07	2038	46	3	1	811	1	1
21_2041	7	2279989	59.58	769059	532	8085892	2032	99.99	0.61	2032	46	3	1	816	1	1
22_10600	17	2258014	59.63	265402	536	8065014	1988	99.99	0.73	1988	46	3	1	772	3	1
22_11067	8	2254011	59.66	789218	538	8077718	1983	100	0.81	1983	46	3	1	757	1	1
22_11177	13	2369525	59.47	575768	120	1902225	2148	99.99	0.77	2163	46	2	1	920	0	0
22_11656	20	2317590	59.8	503698	535	8262610	2069	100	0.53	2069	46	4	1	846	3	1
22_2564	9	2223048	59.67	511382	555	8225546	1961	100	0.13	1961	46	2	1	759	1	1
22_2565	25	2280021	59.51	236242	125	1902225	2028	99.99	0.2	2029	46	3	1	805	2	1
22_2570	4	2245149	59.61	1726273	538	8046760	1990	100	0.16	1990	46	2	1	772	1	1
22_4813	9	2278816	59.63	538847	530	8045136	2019	99.99	0.53	2019	46	4	1	797	2	1
22_4838	15	2267986	59.75	314815	126	1902225	2039	100	0.7	2064	46	3	1	846	2	1
22_5283	6	2230995	59.84	1695154	128	1902225	2012	100	0.38	2043	47	3	1	840	1	1
22_5293	25	2295796	59.58	450692	124	1902225	2037	100	0.83	2223	47	3	1	977	6	1
22_5896	7	2332339	59.45	724224	122	1902225	2085	99.99	0.14	2083	47	3	1	842	1	1
22_7871	25	2290356	59.64	466080	125	1902225	2027	99.99	0.25	2032	47	4	1	808	3	1
23_2752	15	2326575	59.54	518803	123	1902225	2062	100	0.28	2067	47	5	1	850	2	1
23_30761_2	14	2288554	59.45	425539	534	8151654	2022	100	0.72	2022	46	3	1	795	1	1
23_4130	15	2360464	59.53	572355	510	8024512	2153	100	0.84	2153	46	3	1	924	2	1
23_50711_2	18	2350133	59.57	387951	121	1902225	2073	99.99	0.5	2073	46	3	1	828	3	1
23_564_0001_02	17	2245173	59.64	273004	127	1902225	1983	100	0.19	1984	46	3	1	769	2	1
4618_1	15	2326845	59.56	518809	519	8056166	2064	100	0.28	2064	46	3	1	846	3	1
51CBC	19	2246957	59.64	477808	127	1902225	2002	99.99	0.55	2004	46	3	1	781	1	1
93CBB	19	2247703	59.64	290724	127	1902225	2005	99.99	0.55	2005	46	3	1	781	1	1
ATCC_19411	10	2258076	59.56	550533	546	8217662	2002	99.99	0.61	2002	46	2	1	769	1	1

Table S2. Average nucleotide (ANI) of *Trueperella pyogenes* isolates with greater than 99.5% ANI.

Genome 1	Genome 2	ANI	Orthologous fragment count	Total fragment count	ANI %
2022_1_14 (cattle)	315 (cattle)	100	748	749	> 99.99%
2023_3_163 (cattle)	2023_3_174 (cattle)	100	741	742	> 99.99%
51CBC (cattle)	93CBB (cattle)	100	736	739	> 99.99%
2022_1_77 (cattle)	2022_1_29 (cattle)	100	753	755	> 99.99%
GCF_003076315 (swine)	GCF_003076295 (swine)	100	790	794	> 99.99%
GCF_003076315 (swine)	GCF_003971485 (swine)	99.999	791	794	> 99.99%
2022_1_27 (cattle)	306 (cattle)	99.999	748	748	> 99.99%
GCF_003076295 (swine)	GCF_003971485 (swine)	99.999	792	794	> 99.99%
23_2752 (swine)	4618_1 (cattle)	99.996	766	767	> 99.99%
1259 (cattle)	2023_3_174 (cattle)	99.995	741	743	> 99.99%
2023_3_163 (cattle)	1259 (cattle)	99.995	742	742	> 99.99%
2023_3_111 (cattle)	2023_3_175 (cattle)	99.993	723	736	> 99.99%
8896 (swine)	1510 (cattle)	99.992	753	756	> 99.99%
8896 (swine)	23_30761_2 (deer)	99.955	756	756	99.5-99.99%
23_30761_2 (deer)	1510 (cattle)	99.951	753	757	99.5-99.99%
GCF_000612055 (cattle)	GCF_002071825 (cattle)	99.895	668	779	99.5-99.99%
GCF_004009995 (swine)	GCF_004123715 (swine)	99.848	759	775	99.5-99.99%
2022_1_79 (cattle)	306 (cattle)	99.804	742	744	99.5-99.99%
2022_1_27 (cattle)	2022_1_79 (cattle)	99.803	742	748	99.5-99.99%
23_50711_2 (cattle)	2022_1_96 (cattle)	99.8	762	775	99.5-99.99%
2022_1_96 (cattle)	2022_1_30 (cattle)	99.799	765	773	99.5-99.99%
2022_1_96 (cattle)	2023_3_67 (cattle)	99.73	772	773	99.5-99.99%
GCF_004564055 (swine)	4618_1 (cattle)	99.671	738	757	99.5-99.99%
GCF_004564055 (swine)	23_2752 (swine)	99.667	738	757	99.5-99.99%
23_50711_2 (cattle)	2022_1_30 (cattle)	99.636	755	775	99.5-99.99%
1446 (cattle)	23_564_0001_02 (cattle)	99.626	726	739	99.5-99.99%
23_50711_2 (cattle)	2023_3_67 (cattle)	99.621	764	775	99.5-99.99%
2023_3_67 (cattle)	2022_1_30 (cattle)	99.611	765	791	99.5-99.99%
2023_3_60 (cattle)	2023_3_175 (cattle)	99.553	715	723	99.5-99.99%
2023_3_60 (cattle)	2023_3_111 (cattle)	99.544	715	723	99.5-99.99%

Table S3. Distribution and characteristics *Trueperella pyogenes* genotypes based on the presence of virulence genes (n = 83).

Genotype	No. genes	Virulence genes	No. genomes	Isolate IDs
I	7	*plo, fimA, fimC, fimE, nanH, nanP, cbpA*	1	1494
II	6	*plo, fimA, fimC, fimE, nanH, cbpA*	1	UFV1
III	6	*plo, fimA, fimC, fimE, nanH, nanP*	2	22_7871, 22_2565
IV	6	*plo, fimA, fimC, fimE, nanP, cbpA*	5	2023_3_111, 2023_3_175, 2023_3_4, 2023_3_60, 22/KM0800
V	6	*plo, fimA, fimE, nanH, nanP, cbpA*	1	22_2570
VI	5	*plo, fimA, fimC, fimE, cbpA*	6	1446, 2023_3_164, 22_4813, 23_564_0001_02, TP6375, Arash114
VII	5	*plo, fimA, fimC, fimE, nanH*	4	2022_1_14, 22_5293, 315, EMSSI21
VIII	5	*plo, fimA, fimC, fimE, nanP*	12	1355, 1361, 2022_1_75, 2023_3_167, 22_11067, 22_11656, 22_4838, 22_5896, 23_4130, 7762, TP1, TP2
IX	5	*plo, fimA, fimE, nanH, cbpA*	2	51CBC, 93CBB
X	5	*plo, fimA, fimE, nanH, nanP*	1	22_2564
XI	5	*plo, fimA, fimE, nanP, cbpA*	8	1409, 2022_1_30, 2022_1_94, 2022_1_96, 2023_3_67, 23_50711_2, 5861, 09KM1269
XII	4	*fimA, fimC, fimE, nanP*	1	Bu5
XIII	4	*plo, fimA, fimC, fimE*	6	2023_3_104, 2023_3_72, 22_5283, 311, EMSSI54, 13KM1326
XIV	4	*plo, fimA, fimC, nanP*	3	MS249, 22_11177, jx18
XV	4	*plo, fimA, fimE, cbpA*	7	SH01, 2022_1_29, 2022_1_77, 2023_3_71, 2023_4_80, SH02, SH03
XVI	4	*plo, fimA, fimE, nanP*	12	1259, 1510, 2023_3_163, 2023_3_174, 22_10600, 23_2752, 23_30761_2, 4618_1, 8896, TP4479, TP-2849, TP3
XVII	4	*plo, fimC, fimE, nanP*	1	2012CQ-ZSH
XVIII	3	*plo, fimA, fimE*	5	2022_1_27, 2022_1_79, 2023_3_119, 21_2041, 306
XIX	3	*plo, fimA, nanP*	4	2023_3_235, TP4, EMSSI48, 13OD0707
XX	3	*plo, fimC, nanP*	1	ATCC_19411

Table S4. Zone of inhibition (mm) based on the disk diffusion test for *Trueperella pyogenes* isolates (n = 49) to selected antibiotics.

Isolate ID	Animal host	Anatomical body site	Chloramphenicol	Clindamycin	Ciprofloxacin	Sulfa/Trimethoprim	Gentamicin	Penicillin	Tetracycline	Vancomycin	Erythromycin
306	Cattle	Liver abscess	.	.	17	34	22	51	.	29	23
311	Cattle	Liver abscess	32	0	16	40	25	53	18	30	0
315	Cattle	Liver abscess	35	31	16	37	23	52	16	29	44
1259	Cattle	Lung	36	34	17	38	26	53	42	32	47
1355	Sheep	Lung	33	0	14	36	23	50	11	30	0
1409	Cattle	Lung	36	35	22	39	27	52	40	31	49
1446	Cattle	Lung	34	0	15	39	26	43	13	32	0
1494	Deer	Lung	39	36	14	39	26	50	13	29	46
1510	Cattle	Lung	39	34	16	43	26	58	45	31	49
5861	Cattle	Lung	41	28	16	41	26	57	17	32	43
7762	Cattle	Lung	39	35	17	43	29	56	12	33	48
8896	Swine	Lung	34	31	19	39	24	55	42	32	47
164CBD	Cattle	Vaginal swab	38	34	17	38	25	55	13	31	47
2022_1_29	Cattle	Rumen Tissue	33	0	13	36	0	51	11	31	19
2022_1_30	Cattle	Rumen Tissue	33	0	14	35	23	47	12	29	0
2022_1_75	Cattle	Rumen Tissue	34	0	13	36	0	52	11	30	0
2022_1_77	Cattle	Rumen Tissue	36	0	14	33	0	51	11	30	23
2022_1_78	Cattle	Rumen Tissue	32	0	16	36	23	51	11	29	0
2022_1_91	Cattle	Rumen Tissue	15	0	17	38	25	54	12	32	14
2023_3_167	Cattle	Rumen Tissue	37	32	12	37	25	56	39	32	46
2023_3_4	Cattle	Rumen Tissue	38	37	14	33	25	51	13	30	46
2023_3_72	Cattle	Rumen Tissue	35	32	12	36	22	57	12	31	45
2023_4_80	Cattle	Rumen Tissue	34	10	17	40	25	50	12	30	24
2023-3- 235	Cattle	Rumen Tissue	36	32	13	36	26	49	36	29	45
21_2041	Deer	Lung	40	36	18	40	25	54	13	31	48
22_10600	Cat	Nasal swab	41	34	17	42	25	51	18	33	48
22_11067	Ovine	Umbilicus	38	33	17	39	26	54	13	33	45
22_11177	Cattle	Lung	37	33	17	40	25	56	22	31	45
22_11656	Cattle	Lung	39	0	23	36	26	52	14	31	17
22_2564	Cattle	Peri fluid	36	33	17	38	27	52	13	31	46
22_2565	Bison	Lung	41	37	17	39	30	53	44	32	47
22_2570	Cattle	Milk	37	33	18	38	24	54	14	31	45
22_4838	Cattle	Lung	38	35	18	39	27	54	15	32	47
22_5283	Cattle	Lung	37	0	15	40	27	59	13	34	0
22_5293	Cattle	Foot	40	34	18	40	27	58	14	31	46
22_5896	Cattle	Lung	40	34	19	42	25	57	46	32	49
22_7871	Sheep	Mammary	38	34	16	39	25	55	16	31	46
23_2752	Swine	Lung	40	34	19	42	25	60	20	33	53
23_30761_2	Deer	Lung	34	28	17	38	23	56	42	30	44
23_4130	Cattle	Lung	37	0	17	43	28	56	13	32	0
23_564_0001_02	Cattle	Liver	31	0	15	39	25	58	11	32	20
4618_1	Cattle	Abscess	38	33	19	41	25	51	27	32	47
51CBC	Cattle	Vaginal swab	32	31	17	37	24	53	25	30	44
93CBB	Cattle	Vaginal swab	38	32	20	35	28	51	23	31	53
ATCC_19411	Swine	Unknown	38	36	14	38	27	51	46	33	49
2023_3_67	Cattle	Rumen Tissue	32	0	16	37	21	45	9	29	22
2022_1_79	Cattle	Rumen Tissue	31	0	16	34	20	38	11	28	10
2022_1_96	Cattle	Rumen Tissue	37	0	15	35	20	40	10	31	0
23_50711_2	Cattle	Lung	31	0	16	31	17	41	9	30	0

Table S5. Growth of *Trueperella pyogenes* isolates in different carbon sources and chemical assays (n = 49).

Isolate ID	Host	Body site	Chemical	Final OD630	Pos ctrl final OD
23_50711_2	Cattle	Lung	N-Acetyl-D-Glucosamine	0.094	0.508
			D-Serine	0.2	
			D-Fructose	0.311	
			Inosine	0.093	
			Guanidine HCl	0.343	
			Acetoacetic Acid	0.108	
311	Cattle	Liver abscess	Dextrin	0.205	0.407
			D-Salicin	0.533	
2023_3_235	Cattle	Rumen tissue	D-Fructose-6-PO4	0.355	0.516
			D-Malic Acid	0.383	
1510	Cattle	Lung	D-Trehalose	0.467	0.25
			Formic Acid	0.324	
22_4838	Cattle	Lung	Inosine	0.425	0.389
			Mucic Acid	0.449	
22_10600	Cat	Nasal	D-Turanose	0.343	0.598
2022_1_29	Cattle	Rumen tissue	N-Acetyl-D-Glucosamine	0.105	0.402
22_11656	Cattle	Lung	Inosine	0.3	0.317
2022_1_91	Cattle	Rumen tissue	D-Sorbitol	0.447	0.324
23_2752	Swine	Lung	D-Sorbitol	0.282	0.269
2023_4_80	Cattle	Rumen tissue	D-Mannitol	0.45	0.339
23_4130	Cattle	Lung	D-Mannitol	0.386	0.325
2022_1_14	Cattle	Rumen tissue	Glycerol	0.399	0.606
315	Cattle	Liver abscess	Guanidine HCl	0.38	0.549
2022_1_94	Cattle	Rumen tissue	Guanidine HCl	0.225	0.419
2023_3_104	Cattle	Rumen tissue	L-Galactonic Acid Lactone	0.396	0.367
2023_3_72	Cattle	Rumen tissue	D-Saccharic Acid	0.319	0.562
22_5896	Cattle	Lung	Acetoacetic Acid	0.094	0.275

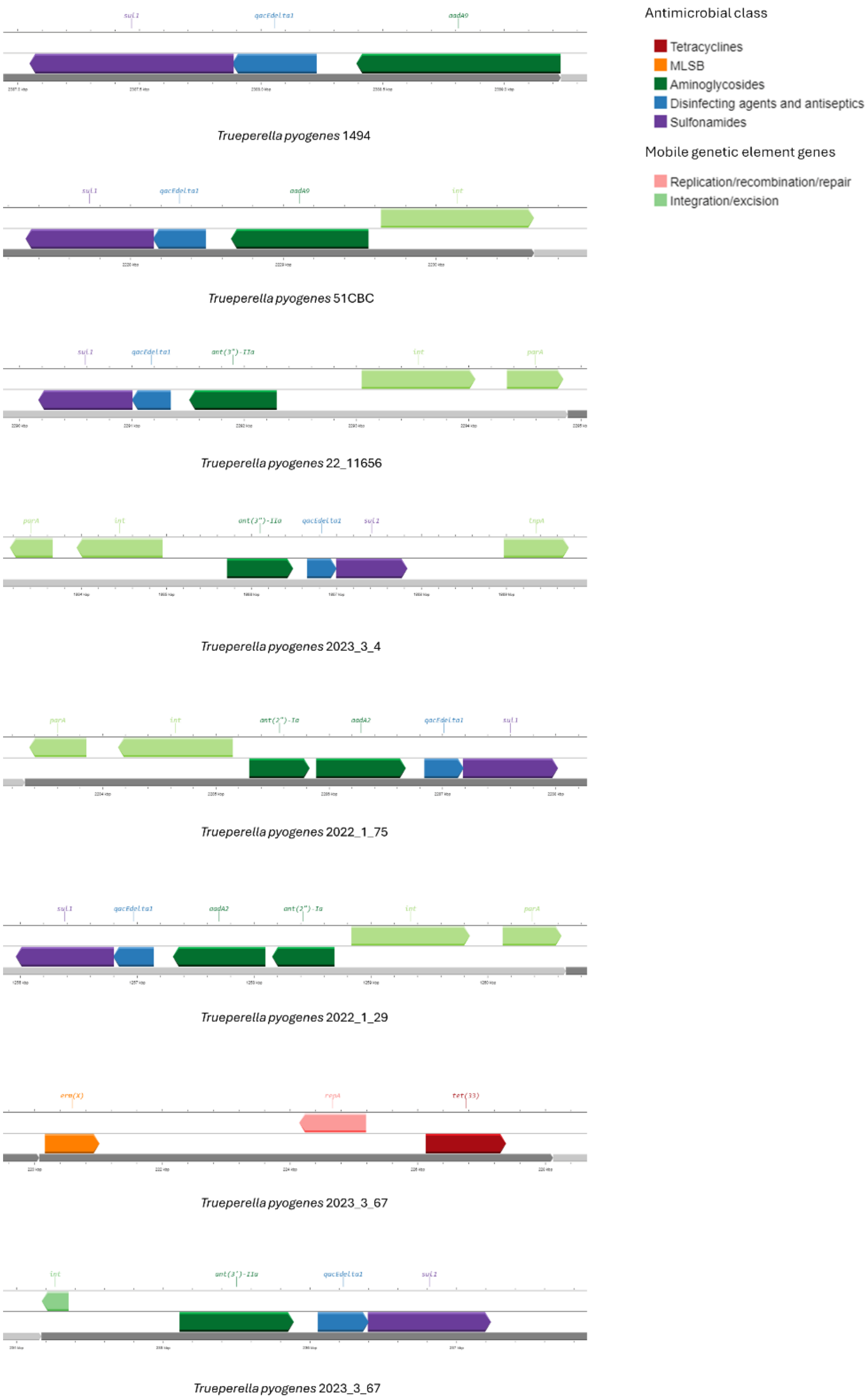

Figure S1. Antimicrobial resistance genes co-located on the same contig in *Trueperella pyogenes* genome assemblies. Also included are mobile genetic element genes.
